# Adolescents' longitudinal trajectories of mental health and loneliness: The impact of COVID‐19 school closures

**DOI:** 10.1002/jad.12017

**Published:** 2022-02-14

**Authors:** Stephen Houghton, Michael Kyron, Simon C. Hunter, David Lawrence, John Hattie, Annemaree Carroll, Corinne Zadow

**Affiliations:** ^1^ Graduate School of Education The University of Western Australia Crawley Western Australia Australia; ^2^ School of Psychological Sciences The University of Western Australia Crawley Western Australia Australia; ^3^ Department of Psychology Glasgow Caledonian University Glasgow UK; ^4^ Graduate School of Education The University of Melbourne Melbourne Victoria Australia; ^5^ Faculty of Humanities and Social Sciences, School of Education The University of Queensland Brisbane Queensland Australia

**Keywords:** COVID‐19, loneliness, longitudinal trajectories, mental health

## Abstract

**Introduction:**

Longitudinal research examining the impact of coronavirus disease 2019 (COVID‐19) school closures on the mental health of adolescents is scarce. Prolonged periods of physical and social isolation because of such restrictions may have impacted heavily on adolescents’ mental health and loneliness.

**Methods:**

The current study addresses a major gap by examining the impact of school closures on the mental health and loneliness of 785, 10‐ to 17‐year‐old Western Australian adolescents (mean age = 14.1, SD = 1.31), who were surveyed across four time points: twice before COVID‐19, once as schools closed, and once post reopening of schools. Pre‐ and post‐COVID‐19 changes in mental health and loneliness were compared using linear mixed models. Random intercept cross‐lagged panel models (RI‐CLPMs) assessed temporal associations between loneliness, depression symptoms, and positive mental wellbeing.

**Results:**

Compared with pre‐COVID‐19 symptom levels, there were significant increases in depression symptoms, internalizing and externalizing symptoms, and a significant decrease in positive mental wellbeing at different points over time. Symptom change over time differed according to gender and pre‐COVID‐19 symptom severity. Significant increases in positive attitudes towards being alone and feelings of isolation occurred at different points over time. Gender differences were evident. RI‐CLPMs highlighted the predictive significance of friendship quality and having a negative attitude towards being alone over time in relation to depression symptoms. A positive or negative attitude towards being alone was predictive of positive mental wellbeing over time.

**Conclusion:**

Findings provide evidence that COVID‐19‐related school closures adversely affected adolescents' mental health and feelings of loneliness.

## 1 INTRODUCTION

Coronavirus disease 2019 (COVID‐19; severe acute respiratory syndrome coronavirus 2) was declared a global pandemic on March 11, 2020, by the World Health Organisation (2020). Within days, the Western Australian (WA) government implemented disease containment measures to slow the potential spread of the virus. On March 24, 2020, the WA government initiated a phased border restriction policy, which within 2 weeks culminated in a “hard border,” preventing persons from other Australian states or international countries entering WA. Already the most isolated capital city in the world, Perth was now effectively completely isolated. Parents were subsequently advised to keep their children at home as schools closed.

Although this had the desired effect of restricting COVID‐19 cases and deaths, the school closures along with home confinement, social distancing, and the cancellation of numerous types of extracurricular activities meant WA adolescents were confronted with prolonged periods of physical and social isolation from friends and family. Existing research shows that the loneliness and social isolation arising from disease containment measures in previous outbreaks (e.g., severe acute respiratory syndrome [SARS] and Middle East respiratory syndrome) were associated with significant increases in risk of mental illness in adolescents and poor mental health up to nine years later (Brooks et al., 2020; Cénat et al., 2020; Loades et al., 2020; Rogers et al., 2020).

There is now mounting evidence globally to suggest that the dramatic changes in response to the COVID‐19 pandemic resulted in poorer general mental health and elevated anxiety and depression levels in adolescents, for example, Asanov et al. (2021) in Ecuador; Branquinho et al. (2020) in Portugal; Chen et al. (2020) in China; Ellis et al. (2020) and Cost et al. (2021) in Canada; Garcia de Avila et al. (2020) in Brazil; Ravens‐Sieber et al. (2021) in Germany; and Yeasmin et al. (2020) in India.

Almost all of the research to date has, however, been cross‐sectional, collected data “during” the pandemic months, and/or relied on retrospective reports. Longitudinal studies that include assessments of mental health before, during, and after COVID‐19 restrictions are scarce (McGinty et al., 2020), as are longitudinal studies including multiple time points during COVID‐19 (Wang et al., 2020). This is particularly the case for longitudinal studies examining the impact of COVID‐19 restrictions on the lives of adolescents. Wang et al. (2020) surveyed the general population in China, including adolescents, on two separate occasions (January 31 to February 2, 2020, ages 16–59 years; and February 28 to March 1, 2020, ages 12–59 years) and found levels of stress, anxiety, and depression remained stable, despite sharp increases in the number of COVID‐19 cases between the two surveys. Although students affected by prolonged school closures reported a higher psychological impact, the participants during the two surveys were not the same.

In Australia, Magson et al. (2021) surveyed the mental health of 248, 13‐ to 16‐year‐olds in the 12 months leading up to the COVID‐19 outbreak and again 2 months following government‐imposed school restrictions. Depressive symptoms and levels of anxiety increased significantly from T1 to T2, whereas life satisfaction decreased significantly. Females experienced greater declines in mental health than males, which is in line with other findings (e.g., Asanov et al., 2021). Similarly, in the United States, reports from *N* = 105 parent–child dyads pointed to increasing trajectories in young adolescent's overall mental health symptoms because of COVID‐19 restrictions (Hussong et al., 2021).

Although the emerging research findings point to a deteriorating situation in adolescents mental health because of COVID‐19 restrictions, there is also evidence that such adverse changes do not remain, and in fact return to pre‐COVID levels once stay‐at‐home orders are lifted (Breaux et al., 2021).

Loneliness, a potential antecedent of emerging mental health issues, has been reported as an unintended consequence of the prolonged periods of physical isolation arising from school closures (Lim et al., 2016). Defined as a subjective and distressing psychological or emotional state, loneliness arises from a perceived deficit in the quality or quantity of an individual's meaningful social relationships (see Cacioppo & Hawkley, 2009; Maes et al., 2016; Matthews et al., 2019; Peplau & Perlman, 1982). Although subjective feelings of loneliness are theoretically distinct from objective social isolation (i.e., number of social contacts/interactions) the two constructs are related (Entringer & Gosling, 2021; Folk et al., 2020) and both are associated with negative health outcomes (Hawkley & Cacioppo, 2010). However, it is the feeling of being connected, rather than the number of social contacts that promotes better health outcomes (Holt‐Lunstad et al., 2010; Luchetti et al., 2020).

The research evidence is unequivocal that feeling lonely is associated with a myriad of mental health and psychosocial problems (e.g., Cacioppo et al., 2002, 2006; Heinrich & Gullone, 2006; Houghton et al., 2016). Furthermore, an increasing trajectory of loneliness during adolescence exacerbates mental health problems (Smith et al., 2018) and predicts self‐harming, suicide ideation, and more lethal suicide attempts (Gvion et al., 2014; Heinrich & Gullone, 2006; Qualter et al., 2013).

Adolescence is already a highly sensitive time of social transformation where there is an increased need for peer interaction and peer friendships (Orben et al., 2020). It is also a time for social comparison (i.e., to gain approval, feel good about themselves, and reduce uncertainty) (Crone & Fuligni, 2020) where peer groups have a significant impact on the socialization of a range of behaviors (Ellis & Zarbatany, 2017). An independent sense of identity is also formed at this time, which can lead to changes in the quantity and quality of personal interactions (Heinrich & Gullone, 2006). COVID‐19‐related school closures and stay‐at‐home orders may therefore have increased risk of feelings of loneliness in young people (Bu et al., 2020; Magson et al., 2021). Studies conducted with adults (age range 18–88 years) have shown that COVID‐19 either led to increased feelings of loneliness (for a review, see Buecker & Horstman, 2021) or had no effect on feelings of loneliness (Bu et al., 2020; Luchetti et al., 2020; Ray, 2021). According to Buecker and Horstmann (2021), the evidence about pandemic containment measures being associated with changes in perceived quality or quantity of social relationships, or both, is inconclusive because studies have not examined these processes.

Deteriorations in adolescent's mental health from prolonged school closures have been reported in longitudinal studies, along with changes in loneliness (Hussong et al., 2021; Magson et al., 2021). For example, utilizing a single loneliness item Li et al. (2021) found over 60% of Australian adolescents “frequently” felt lonely during the COVID‐19 period. “Not feeling connected to friends” was also a key COVID‐19‐related concern of Canadian (Ellis et al., 2020) and Australian adolescents (Li et al., 2021). Given the length of time a person experiences loneliness is a predictor of future mental health problems (Qualter et al., 2010), it is important for educators and policy makers to understand the impact of COVID‐19 school closures.

### 1.1 THE CURRENT STUDY

The research findings so far provide consistent evidence that the COVID‐19 pandemic has had a significant negative impact on adolescent's mental health and, indirectly, their feelings of loneliness. In Australia, where COVID‐19‐related infections and deaths have remained low compared with most other countries, its impact has still been evident. For example, 3 months after disease containment measures were introduced, 75% of 12‐ to 18‐year‐olds across all Australian states reported they were worried about contracting COVID‐19, even though they had little direct or indirect experience with it (Li et al., 2021). Our understanding of the impact of COVID‐19 restrictions on the lives of adolescents’ remains limited due to the scarcity of longitudinal studies, however. Moreover, where evidence has been obtained over time, samples have been relatively small, single items have been used to assess some variables, and the direction of any associations has not been possible due to the timing and/or limited number of assessments undertaken. Although genuine concerns for the mental health of adolescents are warranted, public debate should be informed by reliable data (Koenig et al., 2021).

To address this, the current study presents findings from a four‐wave longitudinal study capturing change in mental health and feelings of loneliness in Australian adolescents before COVID‐19, as schools closed because of COVID‐19, and again following schools reopening. We tested three main hypotheses. First, depression symptoms, externalizing and internalizing symptoms would increase, and positive mental wellbeing would decrease from pre‐COVID to COVID‐19‐related school closures; second, feelings of loneliness would increase from pre‐COVID to COVID‐19‐related school closures, and changes in mental health would be temporally preceded by changes in loneliness; and third when pre‐COVID measures of mental health are averaged and compared with school closures time and post‐COVID schools reopening, there would be a return to pre‐COVID levels. We will examine for sex differences.

## 2 METHODS

### 2.1 Participants and settings

Participants in the current study were part of a larger longitudinal project examining trajectories of loneliness and mental health in adolescents. When COVID‐19 school closures were initiated in March 2020, *N* = 785 adolescents from the larger study had completed Time 3 of the larger project surveys. Of these, 82% (*N* = 644) had completed assessments at Time 1 (pre‐COVID ~18 months earlier) and 93% (*N* = 732) had completed assessments at Time 2 (pre‐COVID ~6 months earlier). When schools reopened (~3 months after Time 3 COVID school closures), 83.95% (*N* = 659) completed the online surveys (i.e., Time 4 schools reopening). These data indicate that participation rates and engagement with the surveys was high over time. The full demographic characteristics at the commencement of school closures (T3) are outlined in Table 1.

**Table 1:** Demographic characteristics of sample

	Frequency or mean	Proportion
Total	785	
Sex		
Male	331	41.2
Female	454	57.8
School year^a^		
Year 7	82	10.5
Year 8	99	12.6
Year 9	245	31.3
Year 10	198	25.3
Year 11	158	20.2
Didn't go to school	1	0.13
Age^a^		
10 Years old	1	0.13
11 Years old	14	1.79
12 Years old	94	12.0
13 Years old	126	16.1
14 Years old	244	31.2
15 Years old	175	224
16 Years old	127	16.2
17 Years old	2	0.26

^a^ Age and school year as at March 2020 (commencement of lockdown).

The participants for the current study were the *N* = 785 adolescents (mean age 14.10 years, SD = 1.31, range = 10–17 years), who had completed the T3 survey in the time leading up to COVID‐19 school closures. These came from seven randomly selected schools (five state government schools and two nongovernment schools) in Perth, Western Australia. These schools were located across a range of socioeconomic status areas as indicated by their Index of Community Socio‐Educational Advantage (ICSEA). ICSEA is set at an average of 1000 (SD = 100) and higher ICSEA values indicate higher levels of educational advantage of students attending the school. The ICSEA values for schools in the study ranged from 939 to 1191.

### 2.2 Measures

#### 2.2.1 Loneliness

The Perth A‐loneness scale (PALs: Houghton et al., 2014) was used to measure adolescent loneliness. This validated 24‐item self‐report measure is composed of four correlated factors, each with six items. Factor 1 measures quality of friendships (e.g., “My friends will stand by me in almost any difficulty”). Factor 2, feelings of isolation (e.g., “I feel like I do not have a friend in the world”). Factor 3, positive attitudes towards being alone (e.g., “I have discovered the benefits of being alone”) and Factor 4, negative attitudes towards being alone (e.g., “When I am all by myself, I wish I had a friend to be with”). Participants respond using a six‐point Likert scale: 1 = never, 2 = rarely, 3 = sometimes, 4 = often, 5 = very often, and 6 = always. In the present study, the quality of friendships (T1–T4, *α*​​​​​​*​* = .90–.93), isolation (T1–T4 *α* = .79–.89), positive attitudes towards being alone (T1–T4 *α* = .85–.88), and negative attitudes towards being alone (T1–T4 *α* = .78–.81) subscales showed good internal consistencies.

### 2.3 Depressive symptoms

The Children's Depression Inventory–2 (self‐report short version; CDI:SR [S] 2; Kovacs, 2004) is a brief self‐report assessment of cognitive, affective, and behavioral symptoms of depression in children and adolescents aged 7–17 years. The CDI:SR [S] 2 is composed of 12 items, each with three separate sentence response options that describe participants’ feelings and ideas over the past 2 weeks. Each item is measured on a three‐point Likert scale, with higher scores indicating poorer outcomes (e.g., 0 = I am sad once in a while, 1 = I am sad many times, 2 = I am sad all the time). The CDI:SR [S] 2 has demonstrated good reliability and discriminant and convergent validity (Kovacs, 2004). In the present study, the CDI:SR [S] 2 had good internal consistency from T1 to T4 (*α* = .83–.86). One item was removed before calculating scores due to content relating to feelings of loneliness which may bias associations with the PALs: Item 12 (“I do not feel alone, I feel alone many times, I feel alone all the time”).

### 2.4 Positive mental wellbeing

The Warwick–Edinburgh Mental Well Being Scale (WEMWBS; Tennant et al., 2007) comprises 14 positively worded items (e.g., “I've been feeling cheerful” and “I've been feeling optimistic about the future”) to which participants respond using a five‐point Likert scale (1 = none of the time, 5 = all of the time). Responses are based on participants’ feelings over the previous 2 weeks. Higher scores indicate higher levels of positive mental wellbeing. In this present study, the WEMWBS had good internal consistency from T1 to T4 (*α* = .83–.87). One item was removed before calculation of total scores due to content relating to feelings of loneliness: “I've been feeling close to other people.”

### 2.5 Internalizing and externalizing symptoms

The Strengths and Difficulties Questionnaire (SDQ; Goodman, 1997) has been used extensively as a screening measure for emotional and behavioral difficulties in children and adolescents (Goodman, 1997). Although the SDQ is designed to produce five subscales (i.e., emotional problems, conduct problems, hyperactivity, peer relationship problems, and prosocial behavior), a three‐subscale division is recommended when assessing nonclinical populations (Goodman et al., 2010). Adolescents self‐reported symptoms on a three‐point Likert scale (0 = almost never, 1 = sometimes, 2 = often), with more significant internalizing and externalizing symptoms indicated by higher scores. In the current study, the internalizing items exhibited acceptable internal consistency across T1–T4 (*α* = .73–.88) and the externalizing items exhibited acceptable‐good internal consistency from T1–T4 (*α* = .78–.80).

### 2.6 Procedure

Permission to conduct the research was obtained from the Human Research Ethics Committee of the administering institution, the WA State Department of Education, and the principals of all participating schools. Permission was also granted by the publisher of the CDI:SR [S] 2 to administer the instrument online. Informed consent and verbal assent was obtained from individual participants. Participants completed the surveys online during regular school time on four separate occasions over the span of ~28 months. Each of the T1–T4 survey administrations remained open for schools and participants to access for a period of 30 days. All participants were provided with a unique identification code, which allowed them to log on to the survey at each separate administration. To ensure that the correct code was used, it was given to each participant immediately before each administration. This unique code also ensured that all information provided was confidential and that data could be linked across waves via these codes for the purposes of data analysis. School principals nominated one teacher to be responsible for liaising with the researchers and overseeing survey administrations across time points. These teachers each received written instructions to ensure standardization of administration procedures. No money or other incentives were provided to participants.

### 2.7 Data analysis

#### 2.7.1 Assessing change in symptoms and loneliness over time

To examine change in symptoms of mental health over time, mixed effects models were fit to the data (using the mixed procedure in SAS Version 9.4). These models assessed whether symptoms of depression, internalizing and externalizing symptoms, positive mental wellbeing, and the four dimensions of loneliness significantly changed over time when compared with pre‐COVID‐19 school closure levels. Depressive symptoms, externalizing and internalizing symptoms, positive mental wellbeing, and the four dimensions of loneliness were modeled linearly, and time was treated as a categorical variable. Both pre‐COVID‐19 time points (T1 and T2) were included in models and T2 was selected as a reference point, as it assessed mental health closest in time to the onset of COVID‐19. Separate mixed effects models were fitted for each variable, with random intercepts and slopes included to account for individual variability. Missing data were handled with maximum likelihood, which has been shown to produce largely equivalent results when compared with multiple imputation (Newman, 2003).

Separate follow‐up models were fitted for males and females to assess whether significant change was evidenced for either genders. During childhood and adolescence, well‐established gender differences exist for mental health and this gap increases with age during adolescence (Van Droogenbroeck et al., 2018). For example, girls report significantly more internalizing disorders (e.g., depression and anxiety) (Campbell et al., 2021), whereas boys present with more externalizing disorders (Plenty et al., 2021). A recent systematic review of studies during the COVID‐19 pandemic showed females were more affected than males for major depressive disorder and anxiety disorders during this period (Santomauro et al., 2021). Despite this evidence, knowledge about gender differences in adolescent mental health remains poorly understood (Campbell et al., 2021). Mean change over time for both genders is depicted graphically and full results from individual growth models are provided in the Supporting Material.

In addition, trajectories over time were examined based on pre‐COVID‐19 T1 and T2. For depression symptoms, categories were developed with reference to normative levels outlined in the CDI:SR [S] 2 (Kovacs, 2004). For positive mental wellbeing, and internalizing and externalizing symptoms, a quartile split was used based on sample distributions. Subsequent mixed effects models assessing the magnitude of change added symptom categories at Time 1 as an independent variable, and change over time assessed with Time 2 as a reference point. Effect sizes have been calculated based on the within‐subjects pooled SD and the magnitude of effects interpreted based on Cohen's guidelines (i.e., weak = 0.2, medium = 0.5, strong = 0.8; Lakens, 2013). Gender, depression symptoms, positive mental wellbeing, and internalizing and externalizing symptoms were nonsignificantly associated with missing data at each other time point when compared with the school closure time assessment. However, adolescents who did not complete pre‐COVID T 2 assessment had a marginally higher mean age.

### 2.8 Temporal relationships between loneliness and depression symptoms

Random intercept cross‐lagged panel models (RI‐CLPMs) were fit to the data to assess the temporal associations between dimensions of loneliness and both depression symptoms and positive mental wellbeing. Cross‐lagged panel models are advantageous in examining reciprocal relationships between variables over time (Usami et al., 2019). Although CLPMs attempt to control for stability in an outcome through autoregressive parameters (i.e., the extent to which one variable is associated with itself at the next assessment), critics suggest this fails to accommodate for the trait‐like, time‐invariant nature of a variable (Hamaker et al., 2015). As a result, the lagged parameters that are obtained with standard CLPMs do not represent the actual within‐person relationships over time and this may lead to erroneous conclusions regarding the presence, predominance, and sign of causal influences. Through the introduction of a random intercept, the RI‐CLPMs allow for examination of cross‐lagged effects and autoregressive components, whereas adequately controlling for stability over time. RI‐CLPMs are advantageous in long‐term longitudinal studies in examining within‐person dynamics. Due to complexity, RI‐CLPM models were fit separately for each subscale of the PALs in relation to depression symptoms or positive mental wellbeing (eight models in total). All four time points were included in each model, but we have omitted the associations between T1 and T2 from the presented results to simplify the diagrams, as these changes were not relevant to our hypotheses. With this approach, scores are interpreted with reference to a person's own mean averaged over time, rather than the overall mean for all participants. This person‐centered approach allows for examination of whether deviations in a factor from typical levels at one timepoint (i.e., higher than average isolation at pre‐COVID) predict change at the next time point (i.e., higher than average depression symptoms at school closures, when controlling for higher than average levels at pre‐COVID). This within‐person approach was utilized to examine whether increases in loneliness from typical levels at the start of COVID‐19 lockdowns were associated with deterioration in mental health at future timepoints. Cross‐sectional correlations between factors were constrained to be equal at each time examining whether deviations from a person‐specific mean in depression symptoms or positive mental wellbeing were correlated with deviations in loneliness. Variables with significant univariate associations with depression or positive mental wellbeing were included in a final model to identify the unique effects of each factor. Full model results have been presented in the Supporting Material (Tables S6–S13).

Results from standard CLPMs have also been presented in Figures S3–S8. These analyses examine levels of loneliness or mental health relative to a grand or overall mean, rather than a person‐specific mean. Within this context, autoregressive associations examine whether deviations in scores from the grand‐mean persisted from one time period to the next (i.e., if an adolescent had depression symptoms higher than the overall average at one time period, how much of this variance persisted at the next time period?). On the other hand, cross‐lagged parameters allow for an assessment of whether scores in a construct relative to the grand‐mean are associated with change in another factor at the next time period (i.e., when controlling for autoregressive association). For example, whether higher than average levels of loneliness relative to other adolescents, are associated with heightened levels of depression symptoms at the next time period. All analyses were conducted using Mplus Version 8.4. All correlations and descriptives at each time point have been reported in Tables S1–S4.

## 3 RESULTS

### 3.1 Changes in symptoms over time

Results from linear mixed models assessing change in symptoms over time are reported in Table 2. In comparison with symptom levels at T2, there were significant increases in depression symptoms (*B* = 0.84, 95% confidence interval [CI] = 0.2, 1.68, *p* = .046) at the COVID‐19 school closure period T3 and a significant decrease in positive mental wellbeing (*B* = −0.05, 95% CI = −0.09, −0.01, *p* = .025). Further, there were significant increases in depression symptoms (*B* = 0.97, 95% CI = 0.09, 1.84, *p* = .03) and internalizing symptoms (*B* = 25, 95% CI = 0.01, 0.48, *p* = .040), and a significant decrease in positive mental wellbeing (*B* = −0.08, 95% CI = −0.12, −0.04, *p* < .001), at the post school reopening period T4 when compared with pre‐COVID‐19 T2 levels. There were no significant changes in externalizing symptoms reported at both T3 COVID‐19 school closure and T4 post school reopening assessments. Inspection of effect sizes indicated that for all time points the effects of change in symptoms were small (i.e., ≤0.2) or negligible.

**Table 2:** Mixed effects models assessing change over time for the full sample

	Depression				Wellbeing				Internalizing				Externalizing			
	Estimate	95% CI	*p*	*d*	Estimate	95% CI	*p*	*d*	Estimate	95% CI	*p*	*d*	Estimate	95% CI	*p*	*d*
Time																
Time 1 (pre‐COVID)	0.02	−0.88, 0.89	.969		0.02	−0.02, 0.07	.177		0.17	−0.07, 0.41	.180		0.08	−0.15, 0.31	.502	
Time 2 (pre‐COVID)	Ref			–	Ref			–	Ref			–	Ref			–
School closures	**0.84**	0.02, 1.68	**.046**	**.07**	**−0.05**	−0.09, −0.01	**.025**	**0.08**	0.18	−0.05, 0.40	.119	.07	0.11	−0.10, 0.33	.302	.04
Post schools reopening	**0.97**	0.09, 1.84	**.030**	**.07**	**−0.08**	−0.12, −0.04	**<.001**	**0.13**	**0.25**	0.01, 0.48	**.040**	**.07**	0.23	−0.00, 0.46	.051	.06

	**Friendships**				**Isolation**				**Positive**				**Negative**			
	Estimate	95% CI	*p*	*d*	Estimate		*p*	*d*	Estimate	95% CI	*p*	*d*	Estimate	95% CI	*p*	*d*
Time																
Time 1 (pre‐COVID)	0.25	−.23, 0.72	.298		**0.47**	0.07, 0.86	**.021**		**−0.59**	−1.00, −0.17	**.006**		**0.82**	0.38, 1.25	**<.001**	
Time 2 (pre‐COVID)	Ref			–	Ref			–	Ref			–	Ref			–
School closures	0.05	−.40, 0.50	.825	.03	0.32	−0.05, 0.69	.092	.06	**0.54**	0.15, 0.93	**.007**	**.11**	0.19	−0.21, 0.60	.354	.04
Post schools reopening	−0.11	−.58, 0.36	.657	.02	**0.42**	0.03, 0.81	**.036**	**.07**	**1.23**	0.82, 1.64	**<.001**	**.20**	−0.28	−0.71, 0.14	.192	.06

*Note*: Cohen's *d* represents change over time relative to Time 2 levels.

Abbreviations: CI, confidence interval; COVID, coronavirus disease.

### 3.2 Trajectories by initial severity

**Figure 1:**
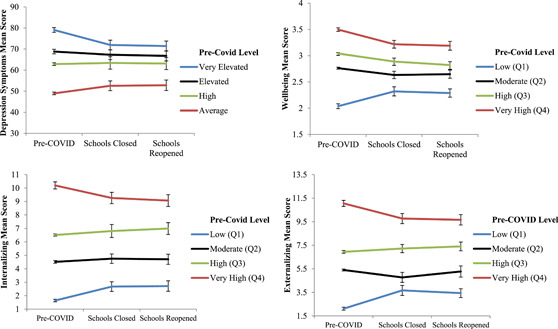
Changes in mental health and loneliness over time by pre‐COVID symptom severity levels. Error bars represent 95% confidence Intervals. COVID, coronavirus disease

Symptom change over time, shown in Figure 1, is based on pre‐COVID‐19 T2 levels. Across each construct, positive changes in depression symptoms, internalizing, and externalizing symptoms were evident for adolescents who had the highest depression symptom levels at pre‐COVID‐19 T2 (i.e., very elevated depression or highest quartile internalizing and externalizing symptoms). In addition, individuals low in positive mental wellbeing at pre‐COVID‐19 T2 reported a higher increase in symptoms compared with those high in positive mental wellbeing at pre‐COVID‐19 T2. Mixed effects models confirmed these differences in trajectories, with changes in depression symptoms (*B* = −3.35, 95% CI = −5.63, −1.07, *p* = .004) and internalizing symptoms (*B* = −1.20, 95% CI = −2.01, −0.38, *p* = .004) by T4 post school reopening being significantly lower for adolescents with higher symptom levels (compared with the lowest symptom category) and higher changes in positive mental wellbeing (*B* = 0.15, 95% CI = 0.01, 0.28, *p* = .034). However, there were no significant changes in externalizing symptoms at T4 post school reopening (*B* = −0.60, 95% CI = −1.28, 0.08, *p* = .081). Effect sizes of the changes in mental health compared to baseline levels based on initial levels have been depicted in Figure S1.

### 3.3 Gender differences

**Figure 2:**
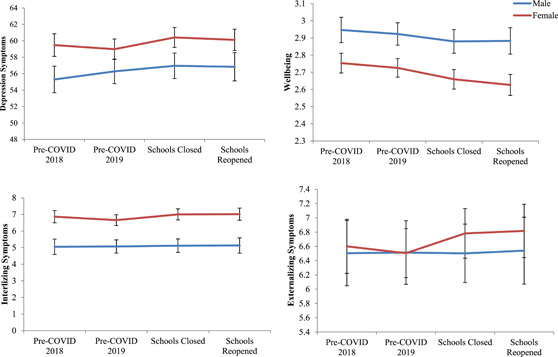
Changes in symptoms over time for male and female adolescents. COVID, coronavirus disease [Color figure can be viewed at wileyonlinelibrary.com]

A gender split was conducted to examine changes over time for males and females (see Figure 2 for the degree of change in symptoms and Table S5 for model results). Results suggested there were nonsignificant changes over time for males in depression symptoms, positive mental wellbeing, externalizing symptoms, and internalizing symptoms. However, for female adolescents there were significant increases in depression symptoms (*B* = 1.29, 95% CI = 0.21, 2.38, *p* = .019) and internalizing symptoms (*B* = 0.32, CI = 0.03, 0.61, *p* = .031), and decreases in positive mental wellbeing (*B* = −0.06, 95% CI = −0.11, *p* = .027) at T3 school closure. At T4 post schools reopening, there were significant increases in depression symptoms (*B* = 1.29, 95% CI = 0.16, 2.42, *p* = .025), externalizing (*β* = .36, 95% CI = 0.06, 0.66, *p* = .018), and internalizing symptoms (*B* = 0.44, CI = 0.14, 0.74, *p* = .004), and decreases in positive mental wellbeing (*B* = −0.11, 95% CI = −0.16, −0.05, *p* < .001). Effect sizes of the changes in mental health compared to baseline levels have been depicted in Figure S2.

### 3.4 Changes in loneliness over time

Compared with pre‐COVID‐19 T2, there was a significant increase in positive attitudes towards being alone at T3 school closure (*B* = 0.54, 95% CI = 0.15, 0.93, *p* = .007) and T4 post school reopening (*B* = 1.23, 95% CI = 0.82, 1.64, *p* < .001). There was a significant increase in feelings of isolation at the T4 post schools reopening (*B* = .42, 95% CI = 0.03, 0.81, *p* = .036), but not at the T3 school closure (*B* = 0.32, 95% CI = −0.05, 0.69, *p* = .092). There were no significant changes in quality of friendships and negative attitudes towards being alone over time.

### 3.5 Gender differences

**Figure 3:**
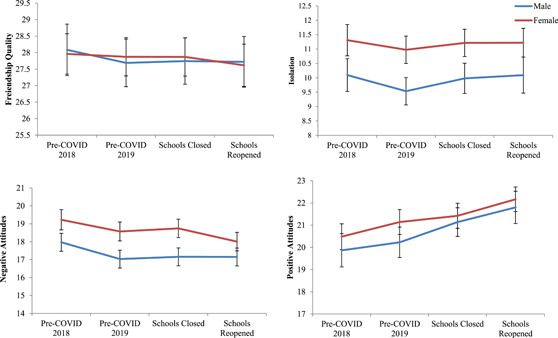
Changes in loneliness over time for male and female adolescents. COVID, coronavirus disease [Color figure can be viewed at wileyonlinelibrary.com]

A gender split was conducted to examine changes over time for males and females (see Table S5 for model results and Figure 3 for graphical depiction). Males reported significant increases in positive attitudes towards being alone at both T3 school closure (*B* = 0.93, CI = 0.30, 1.56, *p* = .004) and T4 post schools reopening (*B* = 1.50, CI = 0.83, 2.17, *p* < .001). For females, there were significant increases in positive attitudes toward being alone (*B* = 1.03, CI = 0.51, 1.56, *p* < .001) and decreases in negative attitudes towards being alone (*B* = −0.57, CI = −1.10, −0.03, *p* = .040) at T4 post schools reopening when compared with pre‐COVID‐19 T2 levels. No significant changes over time in isolation or quality of friendships were evident for either gender.

### 3.6 Temporal associations between loneliness and mental health

**Figure 4:**
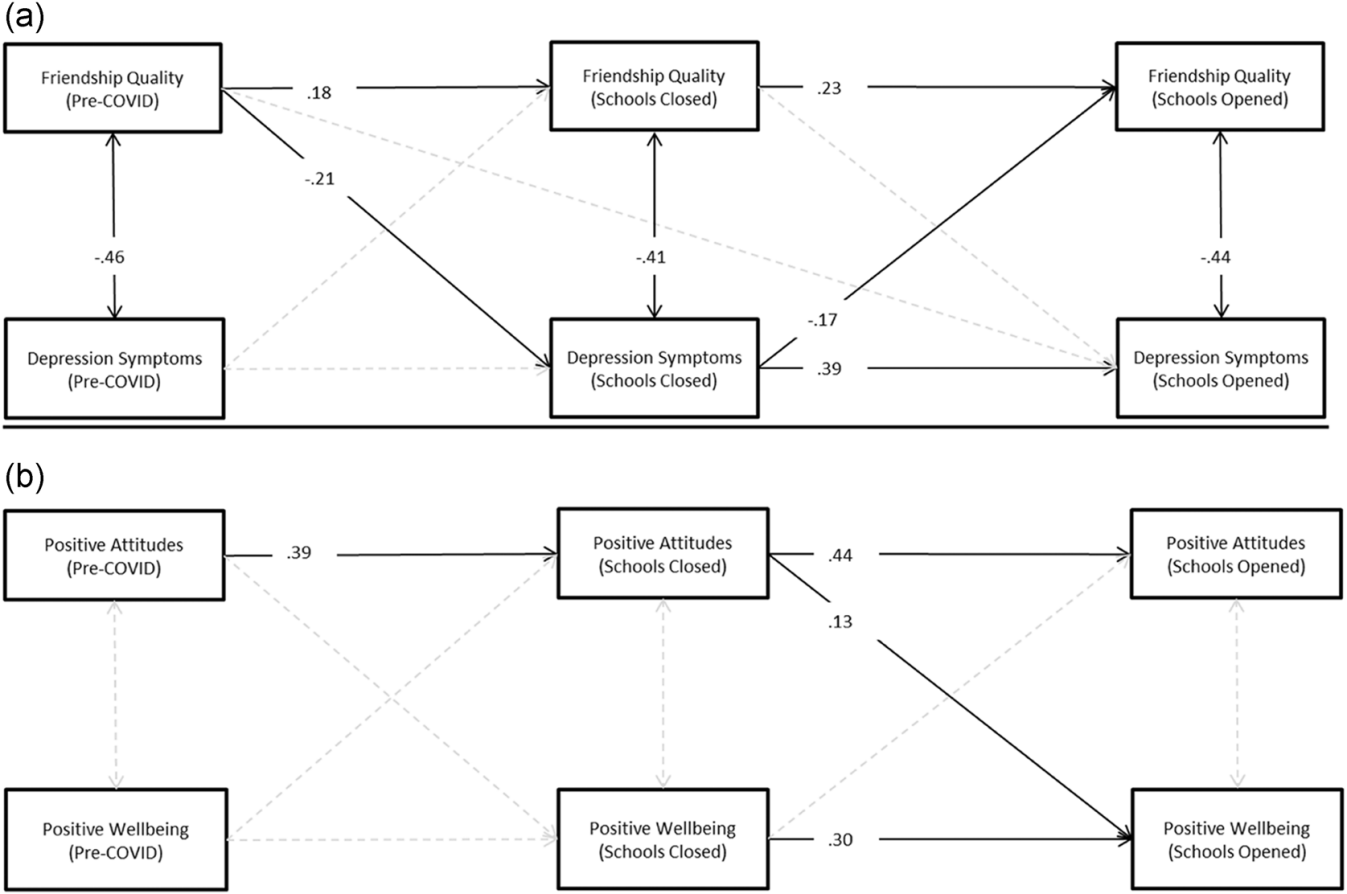
Final random intercept cross‐lagged panel models assessing longitudinal associations between variables. (a) Standardized associations between loneliness and depression symptoms, and (b) associations with wellbeing. Significant cross‐lagged and autoregressive associations have been displayed with black lines for interpretation purposes and nonsignificant associations with dotted gray lines

RI‐CLPMs were fit for each dimension of loneliness to examine within‐level temporal associations with depression symptoms and positive mental wellbeing (Figure 4). Higher person‐centered quality of friendships at pre‐COVID‐19 T2 was significantly associated with lower depression symptoms at T3 when schools closed (*β*​​​​​​ = −.21, 95% CI = −0.34, 0.07, *p* = .015). Feelings of isolation and positive attitudes and negative attitudes toward being alone were non‐significantly associated with depression symptoms over time. Fit to the data for the model with quality of friendships (root mean square of approximation [RMSEA] = 0.04, comparative fit index [CFI] = 0.99, Tucker‐Lewis index [TLI] = 0.98) was excellent.

Higher levels of positive attitudes toward being alone at T3 school lockdowns were associated with higher positive mental wellbeing at T4 when schools reopened (*β* = −.13, *p* = .024). Quality of friendships, isolation, and negative attitudes towards being alone were non‐significantly associated with positive mental wellbeing over time. Fit to the data for the model with positive attitudes toward being alone (RMSEA = 0.03, CFI = 1.00, TLI = .99), was excellent.

Cross‐lagged panel models examined the between‐level associations between loneliness and mental health. Quality of friendships and negative attitudes toward being alone were significantly associated with depression symptoms over the study period. In the final model, quality of friendships at pre‐COVID T2 was significantly associated with lower depression symptoms at T3 school lockdowns (*β* = −.08, 95% CI = −0.13, −0.03, *p* = .036). Higher negative attitudes toward being alone at T2 pre‐COVID was significantly associated with increases in depression symptoms at T3 school lockdowns (*β* = .06, 95% CI = 0.01, 0.11, *p* = .036) and higher negative attitudes toward being alone at T3 school lockdowns was significantly associated with higher depression symptoms at T4 school reopening (*β* = .10, 95% CI = 0.05, 0.15, *p* = .001).

All variables exhibited significant univariate associations with positive mental wellbeing at future time points. In the final model, only higher positive attitudes toward being alone at T2 pre‐COVID was significantly associated with higher positive mental wellbeing at T3 school closures (*β* = .07, 95% CI = 0.00, 0.12, *p* = .040). Higher negative attitudes toward being alone at T3 school closures was significantly associated with lower positive mental wellbeing at T4 school reopenings (*β* = −.09, 95% CI = −0.17, −0.14, *p* = .010).

## 4 DISCUSSION

To date, few longitudinal studies have examined the impact of COVID‐19‐related school closures on the mental health of adolescents. Rather, the emerging research evidence has been primarily cross‐sectional and relied on parents retrospective reporting of pre‐COVID‐19 functioning. The findings from these studies consistently suggest deteriorating mental health among adolescents because of COVID‐19 (e.g., Asanov et al., 2021; Chen et al., 2020; Ellis et al., 2020; Tang et al., 2021; Xie et al., 2020). The limited number of prospective longitudinal studies to date (Breaux et al., 2021; Hussong et al., 2021; Magson et al., 2021) confirm these cross‐sectional study findings. Our current longitudinal research strengthens the existing findings by demonstrating increasing levels of depression symptoms, internalizing symptoms, feelings of isolation, and decreasing positive mental wellbeing among adolescents at different points in time in comparison to their pre‐COVID‐19 school closure levels. In addition, our findings show that lesser increases in symptoms were evident for those with higher initial symptom severity and greater for those low in initial severity. However, it is important to note that those who were among the lowest in initial severity levels still remained within average levels according to normative criteria, and mean changes overall were low. This suggests that adolescents are fundamentally resilient to the adversity associated with COVID‐19 imposed school closures, including the uncertainty and widespread disruptions to daily life.

Magson et al. (2021) investigated whether mental health outcomes from pre to “intra‐COVID‐19” pandemic times differed according to sex. Our findings are supportive of Magson et al. (2021) in that although males evidenced no significant change in depression symptoms, externalizing symptoms, internalizing symptoms, and positive mental wellbeing over time, females did at both commencement of school closures and post schools reopening. In general, girls exhibit more internalizing problems, especially symptoms of depression, compared with boys during adolescence (Campbell et al., 2021; Hankin & Abramson, 1999; Hankin et al., 2007). However, our findings are also supportive of Santomauro et al. (2021) whose systematic review of studies found females were more affected than males for depressive disorder during the COVID‐19 pandemic, and Ravens‐Sieber et al. (2021) who found emotional problems in girls seemed to increase with age during the COVID‐19 pandemic period.

According to Hankin et al. (2007), sex differences in depression are partially explained by girls reporting more “fateful” events outside of their own control, compared with boys. The COVID‐19 school lockdowns and restrictions on social interactions may have been viewed as out of “their” control by the girls in our study and, therefore, although the current findings reinforce the need to monitor the mental health of adolescents into the future, this might be especially important for females.

Stressful life events and social isolation are known to be highly influential factors for externalizing disorders and this may be why, contrary to established research (see Plenty et al., 2021), girls reported more externalizing symptoms than boys at T3 and T4 school lockdown and reopening. Females tend to make plans with nonfamily members more often than males do and have greater quality of friendships (de Jong Gierveld & Van Tilburg, 2010; Houghton et al., 2014). School lockdowns may have disrupted these events to a greater extent than in males, resulting in females giving angrier, inattentive, or impulsive externalizing‐type responses to life situations. Alternatively, as Lancefield et al. (2016) highlighted, it may have been the interaction between internalizing and externalizing psychopathology that explains the results pertaining to girls. For example, externalizing psychopathology is characterized by disinhibition, which affects attentional processing and impulse control. As a result, an individual finds it difficult “to appraise external stimuli appropriately and hold in mind alternative explanations for their occurrence, thereby amplifying cognitive dysfunction” (Lancefield et al., 2016, p. 533).

The impact of COVID‐19 school closures on adolescent's feelings of loneliness has received scant attention in COVID‐19 studies. Li et al. (2021) utilized a single item to measure loneliness “over the past 2 weeks,” but data were collected during the final week of June 2020. Nevertheless, over 50% of respondents still reported frequent feelings of loneliness.

In general, the emerging evidence pertaining to the COVID‐19 pandemic is that feeling connected to friends was important in mitigating the effects of lockdown protocols (Ellis et al., 2020; Magson et al., 2021). Although having this “connection” may be important, it is also the quality of friendships that matter, especially during adolescence (Qualter et al., 2015). This may be particularly important for adolescent girls' because their friendships are characterized by greater levels of intimacy and emotional support (Rose & Rudolph, 2006; Rose, 2002). In the context of disease containment measures, any associated loneliness and social isolation significantly increases risk of mental illness in adolescents (see Loades et al., 2020). In the present study, having reliable, trustworthy, supportive friends was found to be protective against developing depression symptoms at the commencement of school closures. Moreover, having quality friendships pre‐COVID‐19 school closure was predictive of higher levels of positive mental wellbeing when schools closed. These findings are important since the absence of friends can lead to feelings of loneliness, higher rates of morbidity and mortality (Holt‐Lunstad et al., 2010), and long‐term psychopathology (Prinstein et al., 2009).

That feelings of isolation were elevated before school lockdowns (T2) might reflect normal trends, because up to 80% of young people typically report experiencing loneliness (Hawkley & Cacioppo, 2010), while one in ten report “often” feeling lonely (Snape & Manclossi, 2018). However, feelings of isolation increased significantly at the T4 post schools reopening, a time when one might have anticipated such feelings declining due to adolescents being reunited with their peers. Loneliness involves negative cognitions about, for example, relationships, achievement, social acceptance, appearance, the future, upcoming stressful events, and changes in circumstances (Hunter et al., 2021; Songco et al., 2020). Despite young people's efforts to re‐engage in positive ways with their peers at T4, their negative social cognitions about current and further potential COVID‐19 school lockdowns may have exacerbated their feelings of isolation.

It is known that “attitudes towards being alone might affect one's vulnerability to feeling lonely, when alone” (Goossens et al., 2009, p. 890), and that some adolescents have an affinity for being alone while others tend to have an aversion to it (Marcoen et al., 1987). Our findings reveal that having a more positive attitude toward being alone relative to other adolescents at pre‐COVID was associated with higher positive mental wellbeing at T3 school lockdowns, and within‐level increases in positive attitudes toward being alone at the T3 school lockdown assessment was beneficial to adolescents as shown by T4 post school reopening increases in positive mental wellbeing. Conversely, having a higher negative attitude toward being alone relative to others at pre‐COVID was associated with increased depression symptoms at T3 school lockdowns; there were also associations with decreases in positive mental wellbeing. While spending occasional time away from others predicts psychological wellbeing, the key to positive mental health is the ability to enjoy these solitary activities (Leary et al., 2003). Enjoying solitary activities can provide adolescents with pleasurable positive opportunities to become more thoughtful and reflective (Leary et al., 2003). The inevitable disruption to daily functioning because of COVID‐19 school closures may have been quickly appraised by adolescents in the present study. Their thoughtful reflection on the emerging global situation and ensuing social isolation via school closures may have contributed to developing positive attitudes toward being alone and the activities they could access.

Although a strength of the present study is that it addresses the absence of longitudinal studies examining the impact of COVID‐19 on adolescents (McGinty et al., 2020; Wang et al., 2020), there are several limitations that must be acknowledged. There were different time lags between data collections. The first two lags were 6–8 months apart, whereas the COVID‐19 commencement of school closures (T3) and COVID‐19 post schools reopening (T4) were 4–6 months apart. Although our study captured the impacts of COVID‐19 on the mental health and feelings of loneliness of adolescents likely caused by the COVID‐19 imposed school closures, it may have missed periods of heightened loneliness and acute changes to mental health at different times during the COVID‐19 period.

Our data were based on adolescents self‐report and therefore may be subject to bias and poor recall. Although the optimal recommended strategy is to use two or more sources (Antshel et al., 2012), self‐report is an effective means of obtaining an accurate insight into the subjective dispositions (such as mental health and loneliness) that can be difficult to obtain from third parties such as parents and teachers (Baldwin & Dadds, 2007; Houghton et al., 2014). Finally, data were obtained from adolescents in Western Australia, where rates of COVID‐19 infections and death were relatively low compared with other countries and, therefore, the results may not be widely generalizable.

Despite the limitations, this current research adds significantly to the existing research regarding the impact of the COVID‐19 pandemic on the mental health of adolescents. Specifically, it demonstrates increasing trajectories in depression symptoms and internalizing symptoms, and feelings of isolation along with a decline in positive mental wellbeing during the COVID‐19 school closures period. Furthermore, the current findings highlight the importance of quality friendships, especially as schools closed and face‐to‐face contact among adolescents was restricted. Our findings have also highlighted key predictors of future mental health among adolescents, along with potential mediators of the effects of loneliness on mental health. These may be important factors to assess during future pandemic imposed restrictions and other events that result in prolonged periods of social isolation.

In conclusion, “the true impact of the COVID‐19 pandemic on mental health is yet to be seen” (Wickens et al., 2021, p.107). Research following the SARS outbreak found that longitudinal declines in mental health were still ongoing after three years (Wu et al., 2009). It is therefore imperative that further longitudinal studies are conducted, not only to gain a comprehensive understanding of the complexities such events present, but also to develop more targeted and innovative strategies to address the mental health needs of adolescents as they progress into adulthood in uncertain times.

## CONFLICT OF INTERESTS

The authors declared that there are no conflict of interests.

## ETHICS APPROVAL STATEMENT

This study was reviewed and approved by The University of Western Australia (RA/4/20/1039) and the Western Australian Department of Education (D18/0207029). Participating school principals provided consent. All procedures performed in the study involving human participants were in accordance with the ethical standards of the 1975 Helsinki Declaration and its later amendments or comparable ethical standards.

## Supporting information

Supporting information.

Supporting information.

Supporting information.

## Data Availability

The data that support the findings of this study are available on request from the corresponding author. The data are not publicly available due to privacy or ethical restrictions.
