# Supplementary material for: Adolescents' longitudinal trajectories of mental health and loneliness: The impact of COVID‐19 school closures
Source: J Adolesc. 2022 Feb 14;94(2):191–205. doi: 10.1002/jad.12017 (PMC9087620; doi:10.1002/jad.12017)
Supplement: Supplementary file 1 — Supporting information. [file JAD-94-191-s002.docx]

Supplementary Material – Correlation Matrix for Each Time Point

Supplementary Table 1 - Pre-COVID - Time 1

|  | **1).** | **2).** | **3).** | **4).** | **5).** | **6).** | **7).** | **8).** | **Mean** | **SD** | **Min** | **Max** |
| --- | --- | --- | --- | --- | --- | --- | --- | --- | --- | --- | --- | --- |
| **1). Isolation T1** | 1 | - | - | - | - | - | - | - | 10.80 | 5.13 | 6 | 36 |
| **2). Friendship T1** | -0.69*** | 1 | - | - | - | - | - | - | 28.02 | 6.18 | 6 | 36 |
| **3). Positive Attitudes T1** | 0.13*** | -0.15*** | 1 | - | - | - | - | - | 20.26 | 5.93 | 6 | 36 |
| **4). Negative Attitudes T1** | 0.37*** | -0.21*** | -0.34*** | 1 | - | - | - | - | 18.69 | 5.88 | 6 | 36 |
| **5). Depression T1** | 0.58*** | -0.48*** | 0.14*** | 0.27*** | 1 | - | - | - | 57.77 | 13.57 | 40 | 90 |
| **6). Wellbeing T1** | -0.53*** | 0.55*** | -0.09* | -0.20*** | -0.71*** | 1 | - | - | 2.83 | 0.58 | 0.81 | 4.03 |
| **7). Internalizing T1** | 0.62*** | -0.56*** | 0.18*** | 0.29*** | 0.72*** | -0.65*** | - | - | 6.13 | 3.75 | 0 | 19 |
| **8). Externalizing T1** | 0.29*** | -0.29*** | 0.06 | 0.21*** | 0.53*** | -0.51*** | 0.42*** | - | 6.54 | 3.71 | 0 | 19 |

Note * p <.05, ** p <.01, *** p < .001

Supplementary Table 2 - Pre-COVID - Time 2

|  | **1).** | **2).** | **3).** | **4).** | **5).** | **6).** | **7).** | **8).** | **Mean** | **SD** | **Min** | **Max** |
| --- | --- | --- | --- | --- | --- | --- | --- | --- | --- | --- | --- | --- |
| **1). Isolation T2** | 1.00 | - | - | - | - | - |  |  | 10.38 | 4.73 | 6.00 | 30.00 |
| **2). Friendship T2** | -0.68*** | 1.00 | - | - | - | - |  |  | 27.78 | 6.22 | 6.00 | 36.00 |
| **3). Positive Attitudes T2** | 0.14*** | -0.16*** | 1.00 | - | - | - |  |  | 20.75 | 5.98 | 7.00 | 36.00 |
| **4). Negative Attitudes T2** | 0.31*** | -0.15*** | -0.38*** | 1.00 | - | - |  |  | 17.93 | 5.56 | 6.00 | 36.00 |
| **5). Depression T2** | 0.57*** | -0.51*** | 0.12** | 0.27*** | 1.00 | - |  |  | 57.85 | 13.17 | 40.00 | 90.00 |
| **6). Wellbeing T2** | -0.51*** | 0.52*** | -0.08* | -0.22*** | -0.71*** | 1.00 |  |  | 2.81 | 0.58 | 0.81 | 4.03 |
| **7). Internalizing T1** | 0.60*** | -0.53*** | 0.19*** | 0.28*** | 0.68*** | -0.62*** | 1.00 |  | 5.99 | 3.55 | 0 | 17 |
| **8). Externalizing T1** | 0.29*** | -0.31*** | 0.03 | 0.21*** | 0.56*** | -0.48 | 0.43*** | 1.00 | 6.51 | 3.73 | 0 | 17 |

Note * p <.05, ** p <.01, *** p < .001

Supplementary Table 3 - School Lockdown - Time 3

|  | **1).** | **2).** | **3).** | **4).** | **5).** | **6).** | **7).** | **8).** | **Mean** | **SD** | **Min** | **Max** |
| --- | --- | --- | --- | --- | --- | --- | --- | --- | --- | --- | --- | --- |
| **1). Isolation T3** | 1.00 | - | - | - | - | - |  |  | 10.67 | 5.02 | 6 | 36 |
| **2). Friendship T3** | -0.67*** | 1.00 | - | - | - | - |  |  | 27.85 | 6.33 | 6 | 36 |
| **3). Positive Attitudes T3** | 0.09* | -0.04 | 1.00 | - | - | - |  |  | 21.29 | 6.04 | 6 | 36 |
| **4). Negative Attitudes T3** | 0.31*** | -0.10** | -0.32*** | 1.00 | - | - |  |  | 18.05 | 5.67 | 6 | 36 |
| **5). Depression T3** | 0.55*** | -0.53*** | 0.10** | 0.28*** | 1.00 | - |  |  | 58.86 | 13.72 | 40 | 90 |
| **6). Wellbeing T3** | -0.51*** | 0.54*** | -0.07 | -0.23*** | -0.75*** | 1.00 |  |  | 2.76 | 0.62 | 0.81 | 4.03 |
| **7). Internalizing T3** | 0.57*** | -0.55*** | 0.19*** | 0.24*** | 0.67*** | -0.59*** | 1.00 |  | 6.19 | 3.75 | 0 | 18 |
| **8). Externalizing T3** | 0.31*** | -0.28*** | 0.03 | 0.27*** | 0.56*** | -0.42*** | 0.43*** | 1.00 | 6.64 | 3.73 | 0 | 19 |

Note * p <.05, ** p <.01, *** p < .001

Supplementary Table 4 - School Reopening - Time 4

|  | **1).** | **2).** | **3).** | **4).** | **5).** | **6).** | **7).** | **8).** | **Mean** | **SD** | **Min** | **Max** |
| --- | --- | --- | --- | --- | --- | --- | --- | --- | --- | --- | --- | --- |
| **1). Isolation T4** | 1.00 | - | - | - | - | - |  |  | 10.77 | 5.11 | 6 | 36 |
| **2). Friendship T4** | -0.72*** | 1.00 | - | - | - | - |  |  | 27.65 | 6.41 | 7 | 36 |
| **3). Positive Attitudes T4** | 0.10* | -0.07 | 1.00 | - | - | - |  |  | 22.01 | 5.75 | 7 | 36 |
| **4). Negative Attitudes T4** | 0.37*** | -0.21*** | -0.30*** | 1.00 | - | - |  |  | 17.66 | 5.42 | 6 | 36 |
| **5). Depression T4** | 0.57*** | -0.54*** | 0.10* | 0.39*** | 1.00 | - |  |  | 58.75 | 13.62 | 40 | 90 |
| **6). Wellbeing T4** | -0.52*** | 0.56*** | 0.02 | -0.31*** | -0.76*** | 1.00 |  |  | 2.73 | 0.62 | 0.81 | 4.03 |
| **7). Internalizing T4** | 0.61*** | -0.56*** | 0.20*** | 0.31*** | 0.72*** | -0.66*** | 1.00 |  | 6.26 | 3.80 | 0 | 17 |
| **8). Externalizing T4** | 0.33*** | -0.31*** | 0.01 | 0.33 | 0.58*** | -0.48*** | 0.45*** | 1.00 | 6..69 | 3.77 | 0 | 17 |

Note * p <.05, ** p <.01, *** p < .001
