# Supplementary material for: Adolescents' longitudinal trajectories of mental health and loneliness: The impact of COVID‐19 school closures
Source: J Adolesc. 2022 Feb 14;94(2):191–205. doi: 10.1002/jad.12017 (PMC9087620; doi:10.1002/jad.12017)
Supplement: Supplementary file 2 — Supporting information. [file JAD-94-191-s001.docx]

**Supplementary Tables – Estimates from Random Intercept Cross-Lagged Models**

**Supplementary Table 6**

**Friendship – Depression Symptoms**

|  | **Estimate** | **SE** | **Est/S.E.** | ***p*** |
| --- | --- | --- | --- | --- |
| RI_X BY |  |  |  |  |
| DepressionT1 | 0.749 | 0.021 | 35.991 | <.001 |
| DepressionT2 | 0.792 | 0.025 | 32.063 | <.001 |
| DepressionT3 | 0.763 | 0.024 | 31.852 | <.001 |
| DepressionT4 | 0.758 | 0.023 | 32.973 | <.001 |
|  |  |  |  |  |
| RI_Y BY |  |  |  |  |
| FRIENDSHIP_T1 | 0.658 | 0.026 | 25.201 | <.001 |
| FRIENDSHIP_T2 | 0.681 | 0.031 | 21.982 | <.001 |
| FRIENDSHIP_T3 | 0.659 | 0.032 | 20.552 | <.001 |
| FRIENDSHIP_T4 | 0.655 | 0.031 | 21.487 | <.001 |
|  |  |  |  | <.001 |
| CX1 BY |  |  |  |  |
| DepressionT1 | 0.663 | 0.023 | 28.237 | <.001 |
|  |  |  |  |  |
| CX2 BY |  |  |  |  |
| DepressionT2 | 0.61 | 0.032 | 19.026 | <.001 |
|  |  |  |  |  |
| CX3 BY |  |  |  |  |
| DepressionT3 | 0.646 | 0.028 | 22.795 | <.001 |
|  |  |  |  |  |
| CX4 BY |  |  |  |  |
| DepressionT4 | 0.653 | 0.027 | 24.457 | <.001 |
|  |  |  |  |  |
| CY1 BY |  |  |  |  |
| FRIENDSHIP_T1 | 0.753 | 0.023 | 33.009 | <.001 |
|  |  |  |  |  |
| CY2 BY |  |  |  |  |
| FRIENDSHIP_T2 | 0.732 | 0.029 | 25.368 | <.001 |
|  |  |  |  |  |
| CY3 BY |  |  |  |  |
| FRIENDSHIP_T3 | 0.752 | 0.028 | 26.724 | <.001 |
|  |  |  |  |  |
| CY4 BY |  |  |  |  |
| FRIENDSHIP_T4 | 0.755 | 0.026 | 28.522 | <.001 |
|  |  |  |  |  |
| CX2 ON |  |  |  |  |
| CX1 | 0.086 | 0.09 | 0.957 | 0.338 |
| CY1 | 0.024 | 0.069 | 0.347 | 0.729 |
|  |  |  |  |  |
| CX3 ON |  |  |  |  |
| CX2 | 0.069 | 0.106 | 0.654 | 0.513 |
| CY2 | -0.205 | 0.084 | -2.438 | 0.015 |
|  |  |  |  |  |
| CX4 ON |  |  |  |  |
| CX3 | 0.387 | 0.065 | 5.98 | <.001 |
| CY3 | -0.01 | 0.062 | -0.163 | 0.871 |
|  |  |  |  |  |
| CY2 ON |  |  |  |  |
| CX1 | 0.031 | 0.073 | 0.428 | 0.669 |
| CY1 | 0.132 | 0.071 | 1.855 | 0.064 |
|  |  |  |  |  |
| CY3 ON |  |  |  |  |
| CX2 | -0.042 | 0.084 | -0.493 | 0.622 |
| CY2 | 0.18 | 0.089 | 2.032 | 0.042 |
|  |  |  |  |  |
| CY4 ON |  |  |  |  |
| CX3 | -0.166 | 0.069 | -2.408 | 0.016 |
| CY3 | 0.233 | 0.071 | 3.264 | 0.001 |
|  |  |  |  |  |
| CX1 WITH |  |  |  |  |
| CY1 | -0.37 | 0.053 | -6.929 | <.001 |
| RI_X | 0 | 0 | 999 | 999 |
| RI_Y | 0 | 0 | 999 | 999 |
|  |  |  |  |  |
| CX2 WITH |  |  |  |  |
| CY2 | -0.46 | 0.035 | -13.032 | <.001 |
|  |  |  |  |  |
| CX3 WITH |  |  |  |  |
| CY3 | -0.411 | 0.039 | -10.581 | <.001 |
|  |  |  |  |  |
| CX4 WITH |  |  |  |  |
| CY4 | -0.438 | 0.035 | -12.518 | <.001 |
|  |  |  |  |  |
| RI_X WITH |  |  |  |  |
| CY1 | 0 | 0 | 999 | 999 |
| RI_Y | -0.596 | 0.042 | -14.049 | <.001 |
|  |  |  |  |  |
| RI_Y WITH |  |  |  |  |
| CY1 | 0 | 0 | 999 | 999 |

*Note.* SE = Standard Error. *p* = p-value. 999 denotes estimate that cannot be computed.

**Supplementary Table 7**

**Friendship – Positive Mental Wellbeing**

|  | **Estimate** | **SE** | **Est/S.E.** | ***p*** |
| --- | --- | --- | --- | --- |
| RI_X BY |  |  |  |  |
| WB_T1 | 0.713 | 0.023 | 30.552 | < .001 |
| WB_T2 | 0.719 | 0.025 | 28.27 | < .001 |
| WB_T3 | 0.674 | 0.024 | 27.826 | < .001 |
| WB_T4 | 0.676 | 0.023 | 29.085 | < .001 |
|  |  |  |  |  |
| RI_Y BY |  |  |  |  |
| FRIENDSHIP_T1 | 0.651 | 0.025 | 26.369 | < .001 |
| FRIENDSHIP_T2 | 0.664 | 0.028 | 24.013 | < .001 |
| FRIENDSHIP_T3 | 0.65 | 0.028 | 23.441 | < .001 |
| FRIENDSHIP_T4 | 0.652 | 0.028 | 23.484 | < .001 |
|  |  |  |  |  |
| CX1 BY |  |  |  |  |
| WB_T1 | 0.701 | 0.024 | 29.559 | < .001 |
|  |  |  |  |  |
| CX2 BY |  |  |  |  |
| WB_T2 | 0.695 | 0.026 | 26.387 | < .001 |
|  |  |  |  |  |
| CX3 BY |  |  |  |  |
| WB_T3 | 0.739 | 0.022 | 33.437 | < .001 |
|  |  |  |  |  |
| CX4 BY |  |  |  |  |
| WB_T4 | 0.737 | 0.021 | 34.492 | < .001 |
|  |  |  |  |  |
| CY1 BY |  |  |  |  |
| FRIENDSHIP_T1 | 0.759 | 0.021 | 35.937 | < .001 |
|  |  |  |  |  |
| CY2 BY |  |  |  |  |
| FRIENDSHIP_T2 | 0.748 | 0.025 | 30.438 | < .001 |
|  |  |  |  |  |
| CY3 BY |  |  |  |  |
| FRIENDSHIP_T3 | 0.76 | 0.024 | 32.044 | < .001 |
|  |  |  |  |  |
| CY4 BY |  |  |  |  |
| FRIENDSHIP_T4 | 0.758 | 0.024 | 31.702 | < .001 |
|  |  |  |  |  |
| CX2 ON |  |  |  |  |
| CX1 | 0.118 | 0.076 | 1.55 | .121 |
| CY1 | 0.034 | 0.066 | 0.509 | .611 |
|  |  |  |  |  |
| CX3 ON |  |  |  |  |
| CX2 | 0.115 | 0.073 | 1.577 | .115 |
| CY2 | 0.122 | 0.066 | 1.844 | .065 |
|  |  |  |  |  |
| CX4 ON |  |  |  |  |
| CX3 | 0.308 | 0.057 | 5.412 | < .001 |
| CY3 | 0.002 | 0.057 | 0.036 | .972 |
|  |  |  |  |  |
| CY2 ON |  |  |  |  |
| CX1 | 0.093 | 0.066 | 1.412 | 0.158 |
| CY1 | 0.099 | 0.066 | 1.509 | 0.131 |
|  |  |  |  |  |
| CY3 ON |  |  |  |  |
| CX2 | 0.144 | 0.064 | 2.258 | 0.024 |
| CY2 | 0.164 | 0.067 | 2.453 | 0.014 |
|  |  |  |  |  |
| CY4 ON |  |  |  |  |
| CX3 | 0.168 | 0.057 | 2.958 | 0.003 |
| CY3 | 0.239 | 0.058 | 4.094 | < .001 |
|  |  |  |  |  |
| CX1 WITH | |  |  |  |
| CY1 | 0.455 | 0.047 | 9.714 | < .001 |
| RI_X | 0 | 0 | 999 | 999 |
| RI_Y | 0 | 0 | 999 | 999 |
|  |  |  |  |  |
| CX2 WITH | |  |  |  |
| CY2 | 0.456 | 0.032 | 14.355 | < .001 |
|  |  |  |  |  |
| CX3 WITH | |  |  |  |
| CY3 | 0.401 | 0.031 | 13.067 | < .001 |
|  |  |  |  |  |
| CX4 WITH | |  |  |  |
| CY4 | 0.43 | 0.03 | 14.264 | < .001 |
|  |  |  |  |  |
| RI_X WITH | |  |  |  |
| CY1 | 0 | 0 | 999 | 999 |
| RI_Y | 0.638 | 0.038 | 16.681 | < .001 |
|  |  |  |  |  |
| RI_Y WITH | |  |  |  |
| CY1 | 0 | 0 | 999 | 999 |

*Note.* SE = Standard Error. *p* = p-value. 999 denotes estimate that cannot be computed.

**Supplementary Table 8**

**Isolation – Depression Symptoms**

|  | Estimate | SE | Est/S.E. | *p* |
| --- | --- | --- | --- | --- |
| RI_X BY |  |  |  |  |
| DEPRESSIONT1 | 0.747 | 0.02 | 36.456 | < .001 |
| DEPRESSIONT2 | 0.79 | 0.026 | 30.222 | < .001 |
| DEPRESSIONT3 | 0.769 | 0.024 | 31.774 | < .001 |
| DEPRESSIONT4 | 0.765 | 0.024 | 32.289 | < .001 |
|  |  |  |  |  |
| RI_Y BY |  |  |  |  |
| ISOLATION_T1 | 0.607 | 0.032 | 19.083 | < .001 |
| ISOLATION_T2 | 0.672 | 0.034 | 19.906 | < .001 |
| ISOLATION_T3 | 0.643 | 0.036 | 18.029 | < .001 |
| ISOLATION_T4 | 0.625 | 0.038 | 16.594 | < .001 |
|  |  |  |  |  |
| CX1 BY |  |  |  |  |
| DEPRESSIONT1 | 0.665 | 0.023 | 28.892 | < .001 |
|  |  |  |  |  |
| CX2 BY |  |  |  |  |
| DEPRESSIONT2 | 0.614 | 0.034 | 18.25 | < .001 |
|  |  |  |  |  |
| CX3 BY |  |  |  |  |
| DEPRESSIONT3 | 0.639 | 0.029 | 21.93 | < .001 |
|  |  |  |  |  |
| CX4 BY |  |  |  |  |
| DEPRESSIONT4 | 0.644 | 0.028 | 22.941 | < .001 |
|  |  |  |  |  |
| CY1 BY |  |  |  |  |
| ISOLATION_T1 | 0.794 | 0.024 | 32.654 | < .001 |
|  |  |  |  |  |
| CY2 BY |  |  |  |  |
| ISOLATION_T2 | 0.74 | 0.031 | 24.138 | < .001 |
|  |  |  |  |  |
| CY3 BY |  |  |  |  |
| ISOLATION_T3 | 0.766 | 0.03 | 25.552 | < .001 |
|  |  |  |  |  |
| CY4 BY |  |  |  |  |
| ISOLATION_T4 | 0.781 | 0.03 | 25.946 | < .001 |
|  |  |  |  |  |
| CX2 ON |  |  |  |  |
| CX1 | 0.036 | 0.091 | 0.394 | .693 |
| CY1 | 0.111 | 0.074 | 1.508 | .131 |
|  |  |  |  |  |
| CX3 ON |  |  |  |  |
| CX2 | 0.161 | 0.107 | 1.504 | .133 |
| CY2 | -0.024 | 0.074 | -0.324 | .746 |
|  |  |  |  |  |
| CX4 ON |  |  |  |  |
| CX3 | 0.372 | 0.066 | 5.6 | < .001 |
| CY3 | 0.011 | 0.061 | 0.185 | .853 |
|  |  |  |  |  |
| CY2 ON |  |  |  |  |
| CX1 | -0.08 | 0.065 | -1.227 | .220 |
| CY1 | 0.197 | 0.073 | 2.702 | .007 |
|  |  |  |  |  |
| CY3 ON |  |  |  |  |
| CX2 | -0.01 | 0.084 | -0.12 | .904 |
| CY2 | 0.074 | 0.097 | 0.76 | .447 |
|  |  |  |  |  |
| CY4 ON |  |  |  |  |
| CX3 | 0.07 | 0.06 | 1.166 | .244 |
| CY3 | 0.332 | 0.066 | 5.068 | < .001 |
|  |  |  |  |  |
| CX1 WITH | |  |  |  |
| CY1 | 0.499 | 0.05 | 10.064 | < .001 |
| RI_X | 0 | 0 | 999 | 999 |
| RI_Y | 0 | 0 | 999 | 999 |
|  |  |  |  |  |
| CX2 WITH | |  |  |  |
| CY2 | 0.41 | 0.038 | 10.88 | < .001 |
|  |  |  |  |  |
| CX3 WITH | |  |  |  |
| CY3 | 0.351 | 0.035 | 10.017 | < .001 |
|  |  |  |  |  |
| CX4 WITH | |  |  |  |
| CY4 | 0.376 | 0.039 | 9.709 | < .001 |
|  |  |  |  |  |
| RI_X WITH | |  |  |  |
| CY1 | 0 | 0 | 999 | 999 |
| RI_Y | 0.733 | 0.036 | 20.472 | < .001 |
|  |  |  |  |  |
| RI_Y WITH | |  |  |  |
| CY1 | 0 | 0 | 999 | 999 |

*Note.* SE = Standard Error. *p* = p-value. 999 denotes estimate that cannot be computed.

**Supplementary Table 9**

**Isolation – Positive Mental Wellbeing**

|  | Estimate | SE | Est/S.E. | *p* |
| --- | --- | --- | --- | --- |
| RI_X BY |  |  |  |  |
| WB_T1 | 0.72 | 0.023 | 30.702 | < .001 |
| WB_T2 | 0.73 | 0.031 | 23.926 | < .001 |
| WB_T3 | 0.685 | 0.029 | 23.379 | < .001 |
| WB_T4 | 0.684 | 0.029 | 23.496 | < .001 |
|  |  |  |  |  |
| RI_Y BY |  |  |  |  |
| ISOLATION_T1 | 0.603 | 0.032 | 18.948 | < .001 |
| ISOLATION_T2 | 0.662 | 0.035 | 18.736 | < .001 |
| ISOLATION_T3 | 0.639 | 0.037 | 17.178 | < .001 |
| ISOLATION_T4 | 0.623 | 0.038 | 16.187 | < .001 |
|  |  |  |  |  |
| CX1 BY |  |  |  |  |
| WB_T1 | 0.694 | 0.024 | 28.529 | < .001 |
|  |  |  |  |  |
| CX2 BY |  |  |  |  |
| WB_T2 | 0.683 | 0.033 | 20.954 | < .001 |
|  |  |  |  |  |
| CX3 BY |  |  |  |  |
| WB_T3 | 0.729 | 0.028 | 26.504 | < .001 |
|  |  |  |  |  |
| CX4 BY |  |  |  |  |
| WB_T4 | 0.73 | 0.027 | 26.753 | < .001 |
|  |  |  |  |  |
| CY1 BY |  |  |  |  |
| ISOLATION_T1 | 0.797 | 0.024 | 33.093 | < .001 |
|  |  |  |  |  |
| CY2 BY |  |  |  |  |
| ISOLATION_T2 | 0.749 | 0.031 | 24.015 | < .001 |
|  |  |  |  |  |
| CY3 BY |  |  |  |  |
| ISOLATION_T3 | 0.769 | 0.031 | 24.937 | < .001 |
|  |  |  |  |  |
| CY4 BY |  |  |  |  |
| ISOLATION_T4 | 0.783 | 0.031 | 25.565 | < .001 |
|  |  |  |  |  |
| CX2 ON |  |  |  |  |
| CX1 | 0.106 | 0.075 | 1.427 | .154 |
| CY1 | -0.024 | 0.064 | -0.379 | .705 |
|  |  |  |  |  |
| CX3 ON |  |  |  |  |
| CX2 | 0.133 | 0.094 | 1.42 | .156 |
| CY2 | 0.009 | 0.073 | 0.117 | .907 |
|  |  |  |  |  |
| CX4 ON |  |  |  |  |
| CX3 | 0.282 | 0.065 | 4.348 | < .001 |
| CY3 | -0.021 | 0.061 | -0.336 | .737 |
|  |  |  |  |  |
| CY2 ON |  |  |  |  |
| CX1 | 0.029 | 0.067 | 0.439 | .661 |
| CY1 | 0.182 | 0.069 | 2.637 | .008 |
|  |  |  |  |  |
| CY3 ON |  |  |  |  |
| CX2 | -0.021 | 0.082 | -0.259 | .796 |
| CY2 | 0.076 | 0.092 | 0.831 | .406 |
|  |  |  |  |  |
| CY4 ON |  |  |  |  |
| CX3 | -0.107 | 0.061 | -1.765 | .078 |
| CY3 | 0.32 | 0.072 | 4.449 | < .001 |
|  |  |  |  |  |
| CX1 WITH | |  |  |  |
| CY1 | -0.403 | 0.056 | -7.152 | < .001 |
| RI_X | 0 | 0 | 999 | 999 |
| RI_Y | 0 | 0 | 999 | 999 |
|  |  |  |  |  |
| CX2 WITH | |  |  |  |
| CY2 | -0.341 | 0.045 | -7.52 | < .001 |
|  |  |  |  |  |
| CX3 WITH | |  |  |  |
| CY3 | -0.279 | 0.04 | -6.96 | < .001 |
|  |  |  |  |  |
| CX4 WITH | |  |  |  |
| CY4 | -0.296 | 0.05 | -5.936 | < .001 |
|  |  |  |  |  |
| RI_X WITH | |  |  |  |
| CY1 | 0 | 0 | 999 | 999 |
| RI_Y | -0.73 | 0.042 | -17.273 | < .001 |
|  |  |  |  |  |
| RI_Y WITH | |  |  |  |
| CY1 | 0 | 0 | 999 | 999 |
|  |  |  |  |  |
| RI_Y WITH | |  |  |  |
| CY1 0.000 0.000 999.000 999.000 | | | | |

*Note.* SE = Standard Error. *p* = p-value. 999 denotes estimate that cannot be computed.

**Supplementary Table 10**

**Positive Attitudes – Depression Symptoms**

|  | Estimate | SE | Est/S.E. | *p* |
| --- | --- | --- | --- | --- |
| RI_X BY |  |  |  |  |
| DEPRESSIONT1 | 0.751 | 0.021 | 36.375 | <.001 |
| DEPRESSIONT2 | 0.803 | 0.026 | 31.059 | <.001 |
| DEPRESSIONT3 | 0.762 | 0.023 | 32.622 | <.001 |
| DEPRESSIONT4 | 0.759 | 0.022 | 35.089 | <.001 |
|  |  |  |  |  |
| RI_Y BY |  |  |  |  |
| POSATTITUDES_T1 | 0.672 | 0.03 | 22.379 | <.001 |
| POSATTITUDES_T2 | 0.661 | 0.031 | 21.244 | <.001 |
| POSATTITUDES_T3 | 0.654 | 0.031 | 20.82 | <.001 |
| POSATTITUDES_T4 | 0.681 | 0.031 | 21.948 | <.001 |
|  |  |  |  |  |
| CX1 BY |  |  |  |  |
| DEPRESSIONT1 | 0.66 | 0.024 | 28.04 | <.001 |
|  |  |  |  |  |
| CX2 BY |  |  |  |  |
| DEPRESSIONT2 | 0.597 | 0.035 | 17.168 | <.001 |
|  |  |  |  |  |
| CX3 BY |  |  |  |  |
| DEPRESSIONT3 | 0.647 | 0.027 | 23.548 | <.001 |
|  |  |  |  |  |
| CX4 BY |  |  |  |  |
| DEPRESSIONT4 | 0.651 | 0.025 | 25.763 | <.001 |
|  |  |  |  |  |
| CY1 BY |  |  |  |  |
| POSATTITUDES_T1 | 0.74 | 0.027 | 27.127 | <.001 |
|  |  |  |  |  |
| CY2 BY |  |  |  |  |
| POSATTITUDES_T2 | 0.751 | 0.027 | 27.422 | <.001 |
|  |  |  |  |  |
| CY3 BY |  |  |  |  |
| POSATTITUDES_T3 | 0.757 | 0.027 | 27.915 | <.001 |
|  |  |  |  |  |
| CY4 BY |  |  |  |  |
| POSATTITUDES_T4 | 0.732 | 0.029 | 25.333 | <.001 |
|  |  |  |  |  |
| CX2 ON |  |  |  |  |
| CX1 | 0.052 | 0.094 | 0.555 | .579 |
| CY1 | 0.068 | 0.074 | 0.915 | .360 |
|  |  |  |  |  |
| CX3 ON |  |  |  |  |
| CX2 | 0.138 | 0.098 | 1.413 | .158 |
| CY2 | -0.002 | 0.057 | -0.032 | .975 |
|  |  |  |  |  |
| CX4 ON |  |  |  |  |
| CX3 | 0.403 | 0.061 | 6.643 | <.001 |
| CY3 | -0.098 | 0.056 | -1.764 | .078 |
|  |  |  |  |  |
| CY2 ON |  |  |  |  |
| CX1 | -0.068 | 0.059 | -1.162 | .245 |
| CY1 | 0.285 | 0.066 | 4.301 | <.001 |
|  |  |  |  |  |
| CY3 ON |  |  |  |  |
| CX2 | -0.056 | 0.066 | -0.858 | .391 |
| CY2 | 0.382 | 0.058 | 6.605 | <.001 |
|  |  |  |  |  |
| CY4 ON |  |  |  |  |
| CX3 | -0.077 | 0.055 | -1.387 | .166 |
| CY3 | 0.429 | 0.057 | 7.508 | <.001 |
|  |  |  |  |  |
| CX1 WITH | |  |  |  |
| CY1 | 0.097 | 0.066 | 1.47 | .142 |
| RI_X | 0 | 0 | 999 | 999 |
| RI_Y | 0 | 0 | 999 | 999 |
|  |  |  |  |  |
| CX2 WITH | |  |  |  |
| CY2 | 0.031 | 0.038 | 0.804 | .421 |
|  |  |  |  |  |
| CX3 WITH | |  |  |  |
| CY3 | 0.027 | 0.034 | 0.803 | .422 |
|  |  |  |  |  |
| CX4 WITH | |  |  |  |
| CY4 | 0.033 | 0.04 | 0.808 | .419 |
|  |  |  |  |  |
| RI_X WITH | |  |  |  |
| CY1 | 0 | 0 | 999 | 999 |
| RI_Y | 0.189 | 0.058 | 3.235 | .001 |
|  |  |  |  |  |
| RI_Y WITH | |  |  |  |
| CY1 | 0 | 0 | 999 | 999 |

*Note.* SE = Standard Error. *p* = p-value. 999 denotes estimate that cannot be computed.

**Supplementary Table 11**

**Positive Attitudes – Positive Mental Wellbeing**

|  | **Estimate** | **SE** | **Est/S.E.** | ***p*** |
| --- | --- | --- | --- | --- |
| RI_X BY |  |  |  |  |
| WB_T1 | 0.726 | 0.023 | 30.94 | <.001 |
| WB_T2 | 0.748 | 0.03 | 25.296 | <.001 |
| WB_T3 | 0.681 | 0.026 | 25.72 | <.001 |
| WB_T4 | 0.684 | 0.027 | 25.596 | <.001 |
|  |  |  |  |  |
| RI_Y BY |  |  |  |  |
| POSATTITUDES_T1 | 0.665 | 0.032 | 20.491 | <.001 |
| POSATTITUDES_T2 | 0.653 | 0.033 | 19.531 | <.001 |
| POSATTITUDES_T3 | 0.647 | 0.033 | 19.402 | <.001 |
| POSATTITUDES_T4 | 0.672 | 0.033 | 20.652 | <.001 |
|  |  |  |  |  |
| CX1 BY |  |  |  |  |
| WB_T1 | 0.688 | 0.025 | 27.758 | <.001 |
|  |  |  |  |  |
| CX2 BY |  |  |  |  |
| WB_T2 | 0.664 | 0.033 | 19.936 | <.001 |
|  |  |  |  |  |
| CX3 BY |  |  |  |  |
| WB_T3 | 0.733 | 0.025 | 29.814 | <.001 |
|  |  |  |  |  |
| CX4 BY |  |  |  |  |
| WB_T4 | 0.729 | 0.025 | 29.111 | <.001 |
|  |  |  |  |  |
| CY1 BY |  |  |  |  |
| POSATTITUDES_T1 | 0.747 | 0.029 | 25.858 | <.001 |
|  |  |  |  |  |
| CY2 BY |  |  |  |  |
| POSATTITUDES_T2 | 0.757 | 0.029 | 26.267 | <.001 |
|  |  |  |  |  |
| CY3 BY |  |  |  |  |
| POSATTITUDES_T3 | 0.763 | 0.028 | 27.008 | <.001 |
|  |  |  |  |  |
| CY4 BY |  |  |  |  |
| POSATTITUDES_T4 | 0.74 | 0.03 | 25.017 | <.001 |
|  |  |  |  |  |
| CX2 ON |  |  |  |  |
| CX1 | 0.084 | 0.076 | 1.107 | .268 |
| CY1 | -0.019 | 0.072 | -0.257 | .797 |
|  |  |  |  |  |
| CX3 ON |  |  |  |  |
| CX2 | 0.114 | 0.086 | 1.334 | .182 |
| CY2 | 0.011 | 0.062 | 0.181 | .857 |
|  |  |  |  |  |
| CX4 ON |  |  |  |  |
| CX3 | 0.3 | 0.061 | 4.962 | <.001 |
| CY3 | 0.134 | 0.06 | 2.255 | .024 |
|  |  |  |  |  |
| CY2 ON |  |  |  |  |
| CX1 | 0.041 | 0.059 | 0.703 | .482 |
| CY1 | 0.29 | 0.067 | 4.311 | <.001 |
|  |  |  |  |  |
| CY3 ON |  |  |  |  |
| CX2 | -0.052 | 0.061 | -0.857 | .392 |
| CY2 | 0.387 | 0.06 | 6.493 | <.001 |
|  |  |  |  |  |
| CY4 ON |  |  |  |  |
| CX3 | 0.022 | 0.053 | 0.406 | .685 |
| CY3 | 0.438 | 0.057 | 7.719 | <.001 |
|  |  |  |  |  |
| CX1 WITH | |  |  |  |
| CY1 | -0.006 | 0.073 | -0.086 | .931 |
| RI_X | 0 | 0 | 999 | 999 |
| RI_Y | 0 | 0 | 999 | 999 |
|  |  |  |  |  |
| CX2 WITH | |  |  |  |
| CY2 | 0.008 | 0.04 | 0.201 | .841 |
|  |  |  |  |  |
| CX3 WITH | |  |  |  |
| CY3 | 0.007 | 0.034 | 0.201 | .841 |
|  |  |  |  |  |
| CX4 WITH | |  |  |  |
| CY4 | 0.008 | 0.039 | 0.201 | .841 |
|  |  |  |  |  |
| RI_X WITH | |  |  |  |
| CY1 | 0 | 0 | 999 | 999 |
| RI_Y | -0.123 | 0.067 | -1.839 | .066 |
|  |  |  |  |  |
| RI_Y WITH | |  |  |  |
| CY1 | 0 | 0 | 999 | 999 |

*Note.* SE = Standard Error. *p* = p-value. 999 denotes estimate that cannot be computed.

**Supplementary Table 12**

**Negative Attitudes – Depression Symptoms**

|  | **Estimate** | **SE** | **Est/S.E.** | ***p*** |
| --- | --- | --- | --- | --- |
| RI_X BY |  |  |  |  |
| DEPRESSIONT1 | 0.743 | 0.021 | 36.186 | <.001 |
| DEPRESSIONT2 | 0.779 | 0.024 | 33.081 | <.001 |
| DEPRESSIONT3 | 0.751 | 0.023 | 33.199 | <.001 |
| DEPRESSIONT4 | 0.759 | 0.021 | 36.44 | <.001 |
|  |  |  |  |  |
| RI_Y BY |  |  |  |  |
| NEGATTITUDES_T1 | 0.62 | 0.024 | 25.348 | <.001 |
| NEGATTITUDES_T2 | 0.668 | 0.029 | 23.198 | <.001 |
| NEGATTITUDES_T3 | 0.654 | 0.029 | 22.644 | <.001 |
| NEGATTITUDES_T4 | 0.685 | 0.027 | 25.634 | <.001 |
|  |  |  |  |  |
| CX1 BY |  |  |  |  |
| DEPRESSIONT1 | 0.669 | 0.023 | 29.306 | <.001 |
|  |  |  |  |  |
| CX2 BY |  |  |  |  |
| DEPRESSIONT2 | 0.628 | 0.029 | 21.492 | <.001 |
|  |  |  |  |  |
| CX3 BY |  |  |  |  |
| DEPRESSIONT3 | 0.661 | 0.026 | 25.722 | <.001 |
|  |  |  |  |  |
| CX4 BY |  |  |  |  |
| DEPRESSIONT4 | 0.651 | 0.024 | 26.757 | <.001 |
|  |  |  |  |  |
| CY1 BY |  |  |  |  |
| NEGATTITUDES_T1 | 0.785 | 0.019 | 40.62 | <.001 |
|  |  |  |  |  |
| CY2 BY |  |  |  |  |
| NEGATTITUDES_T2 | 0.744 | 0.026 | 28.8 | <.001 |
|  |  |  |  |  |
| CY3 BY |  |  |  |  |
| NEGATTITUDES_T3 | 0.757 | 0.025 | 30.376 | <.001 |
|  |  |  |  |  |
| CY4 BY |  |  |  |  |
| NEGATTITUDES_T4 | 0.728 | 0.025 | 28.936 | <.001 |
|  |  |  |  |  |
| CX2 ON |  |  |  |  |
| CX1 | 0.116 | 0.075 | 1.545 | .122 |
| CY1 | -0.044 | 0.06 | -0.724 | .469 |
|  |  |  |  |  |
| CX3 ON |  |  |  |  |
| CX2 | 0.195 | 0.073 | 2.665 | .008 |
| CY2 | 0.021 | 0.059 | 0.362 | .717 |
|  |  |  |  |  |
| CX4 ON |  |  |  |  |
| CX3 | 0.403 | 0.053 | 7.607 | <.001 |
| CY3 | 0.038 | 0.052 | 0.725 | .468 |
|  |  |  |  |  |
| CY2 ON |  |  |  |  |
| CX1 | -0.052 | 0.057 | -0.928 | .353 |
| CY1 | 0.214 | 0.056 | 3.849 | <.001 |
|  |  |  |  |  |
| CY3 ON |  |  |  |  |
| CX2 | 0.013 | 0.06 | 0.224 | .823 |
| CY2 | 0.162 | 0.066 | 2.466 | .014 |
|  |  |  |  |  |
| CY4 ON |  |  |  |  |
| CX3 | 0.13 | 0.057 | 2.267 | .023 |
| CY3 | 0.25 | 0.059 | 4.238 | <.001 |
|  |  |  |  |  |
| CX1 WITH | |  |  |  |
| CY1 | 0.185 | 0.055 | 3.344 | .001 |
| RI_X | 0 | 0 | 999 | 999 |
| RI_Y | 0 | 0 | 999 | 999 |
|  |  |  |  |  |
| CX2 WITH | |  |  |  |
| CY2 | 0.194 | 0.033 | 5.821 | <.001 |
|  |  |  |  |  |
| CX3 WITH | |  |  |  |
| CY3 | 0.172 | 0.03 | 5.645 | <.001 |
|  |  |  |  |  |
| CX4 WITH | |  |  |  |
| CY4 | 0.214 | 0.038 | 5.584 | <.001 |
|  |  |  |  |  |
| RI_X WITH | |  |  |  |
| CY1 | 0 | 0 | 999 | 999 |
| RI_Y | 0.395 | 0.05 | 7.971 | <.001 |
|  |  |  |  |  |
| RI_Y WITH | |  |  |  |
| CY1 | 0 | 0 | 999 | 999 |

*Note.* SE = Standard Error. *p* = p-value. 999 denotes estimate that cannot be computed.

**Supplementary Table 13**

**Negative Attitudes – Positive Mental Wellbeing**

|  | **Estimate** | **SE** | **Est/S.E.** | ***p*** |
| --- | --- | --- | --- | --- |
| RI_X BY |  |  |  |  |
| Wellbeing_T1 | 0.718 | 0.023 | 30.884 | <.001 |
| Wellbeing_T2 | 0.733 | 0.029 | 25.505 | <.001 |
| Wellbeing_T3 | 0.676 | 0.027 | 24.923 | <.001 |
| Wellbeing_T4 | 0.685 | 0.028 | 24.807 | <.001 |
|  |  |  |  |  |
| RI_Y BY |  |  |  |  |
| NegativeAttitudes_T1 | 0.622 | 0.025 | 25.319 | <.001 |
| NegativeAttitudes_T2 | 0.674 | 0.03 | 22.418 | <.001 |
| NegativeAttitudes_T3 | 0.658 | 0.03 | 21.642 | <.001 |
| NegativeAttitudes_T4 | 0.684 | 0.031 | 22.362 | <.001 |
|  |  |  |  |  |
| CX1 BY |  |  |  |  |
| Wellbeing_T1 | 0.696 | 0.024 | 29.087 | <.001 |
|  |  |  |  |  |
| CX2 BY |  |  |  |  |
| Wellbeing_T2 | 0.681 | 0.031 | 22.015 | <.001 |
|  |  |  |  |  |
| CX3 BY |  |  |  |  |
| Wellbeing_T3 | 0.737 | 0.025 | 29.592 | <.001 |
|  |  |  |  |  |
| CX4 BY |  |  |  |  |
| Wellbeing_T4 | 0.728 | 0.026 | 27.997 | <.001 |
|  |  |  |  |  |
| CY1 BY |  |  |  |  |
| NegativeAttitudes_T1 | 0.783 | 0.02 | 40.069 | <.001 |
|  |  |  |  |  |
| CY2 BY |  |  |  |  |
| NegativeAttitudes_T2 | 0.738 | 0.027 | 26.879 | <.001 |
|  |  |  |  |  |
| CY3 BY |  |  |  |  |
| NegativeAttitudes_T3 | 0.753 | 0.027 | 28.379 | <.001 |
|  |  |  |  |  |
| CY4 BY |  |  |  |  |
| NegativeAttitudes_T4 | 0.729 | 0.029 | 25.405 | <.001 |
|  |  |  |  |  |
| CX2 ON |  |  |  |  |
| CX1 | 0.138 | 0.069 | 1.995 | .046 |
| CY1 | 0.093 | 0.059 | 1.577 | .115 |
|  |  |  |  |  |
| CX3 ON |  |  |  |  |
| CX2 | 0.137 | 0.082 | 1.675 | .094 |
| CY2 | -0.034 | 0.064 | -0.527 | .598 |
|  |  |  |  |  |
| CX4 ON |  |  |  |  |
| CX3 | 0.295 | 0.064 | 4.639 | <.001 |
| CY3 | -0.031 | 0.062 | -0.5 | .617 |
|  |  |  |  |  |
| CY2 ON |  |  |  |  |
| CX1 | 0.057 | 0.059 | 0.967 | .334 |
| CY1 | 0.206 | 0.061 | 3.392 | .001 |
|  |  |  |  |  |
| CY3 ON |  |  |  |  |
| CX2 | 0.051 | 0.06 | 0.847 | .397 |
| CY2 | 0.157 | 0.078 | 2.005 | .045 |
|  |  |  |  |  |
| CY4 ON |  |  |  |  |
| CX3 | -0.104 | 0.062 | -1.679 | .093 |
| CY3 | 0.259 | 0.07 | 3.674 | <.001 |
|  |  |  |  |  |
| CX1 WITH | |  |  |  |
| CY1 | -0.133 | 0.064 | -2.088 | .037 |
| RI_X | 0 | 0 | 999 | 999 |
| RI_Y | 0 | 0 | 999 | 999 |
|  |  |  |  |  |
| CX2 WITH | |  |  |  |
| CY2 | -0.145 | 0.043 | -3.367 | .001 |
|  |  |  |  |  |
| CX3 WITH | |  |  |  |
| CY3 | -0.117 | 0.036 | -3.256 | .001 |
|  |  |  |  |  |
| CX4 WITH | |  |  |  |
| CY4 | -0.138 | 0.044 | -3.1 | .002 |
|  |  |  |  |  |
| RI_X WITH | |  |  |  |
| CY1 | 0 | 0 | 999 | 999 |
| RI_Y | -0.345 | 0.06 | -5.784 | <.001 |
|  |  |  |  |  |
| RI_Y WITH | |  |  |  |
| CY1 | 0 | 0 | 999 | 999 |
| CY1 0.000 0.000 999.000 999.000 | | | | |

*Note.* SE = Standard Error. *p* = p-value. 999 denotes estimate that cannot be computed.
