# Supplementary material for: Adolescents' longitudinal trajectories of mental health and loneliness: The impact of COVID‐19 school closures
Source: J Adolesc. 2022 Feb 14;94(2):191–205. doi: 10.1002/jad.12017 (PMC9087620; doi:10.1002/jad.12017)
Supplement: Supplementary file 3 — Supporting information. [file JAD-94-191-s003.docx]

**Supplementary Table 5**

*Mixed effects models assessing change over time for each gender.*

|  | **Depression** | | | | | | **Wellbeing** | | | | | |
| --- | --- | --- | --- | --- | --- | --- | --- | --- | --- | --- | --- | --- |
|  | *Male* | | | *Female* | | | *Male* | | | *Female* | | |
|  | Estimate | *p* |  | Estimate | *p* |  | Estimate | *p* |  | Estimate | *p* |  |
| Time - |  |  |  |  |  |  |  |  |  |  |  |  |
| Time 1 (Pre-COVID) | -0.70 | .327 |  | 0.48 | .408 |  | 0.02 | .642 |  | 0.03 | .307 |  |
| Time 2 (Pre-COVID) | *(ref)* |  | *-* | *(ref)* |  | *-* | *(ref)* |  | *-* | *(ref)* |  | *-* |
| Schools Closed | **0.23** | **.725** |  | **1.30** | **.019** |  | -0.03 | .551 |  | **-0.06** | **.027** |  |
| Schools Reopened | **0.49** | **.494** |  | **1.29** | **.025** |  | -.04 | .281 |  | **-.11** | **<.001** |  |
|  | **Internalizing** | | | | | | **Externalizing** | | | | | |
|  | *Male* | | | *Female* | | | *Male* | | | *Female* | | |
|  | Estimate | *p* |  | Estimate | *p* | *d* | Estimate | *p* |  | Estimate | *p* |  |
| Time - |  |  |  |  |  |  |  |  |  |  |  |  |
| Time 1 (Pre-COVID) | -0.02 | .935 |  | 0.29 | .061 |  | 0.10 | .595 |  | 0.07 | .638 |  |
| Time 2 (Pre-COVID) | *(ref)* |  | *-* | *(ref)* |  | *-* | *(ref)* |  | *-* | *(ref)* |  | *-* |
| Schools Closed | -0.02 | .903 |  | **0.32** | **.031** |  | -0.07 | .652 |  | 0.25 | .090 |  |
| Schools Reopened | -0.06 | .744 |  | **0.44** | **.004** |  | 0.02 | .900 |  | 0.36 | .018 |  |
|  | **Friendship** | | | | | | **Isolation** | | | | | |
|  | *Male* | | | *Female* | | | *Male* | | | *Female* | | |
|  | Estimate | *p* |  | Estimate | *p* |  | Estimate | *p* | *d* | Estimate | *p* |  |
| Time - |  |  |  |  |  |  |  |  |  |  |  |  |
| Time 1 (Pre-COVID) | 0.48 | .202 |  | 0.11 | .743 |  | 0.02 | .642 |  | 0.62 | .042 |  |
| Time 2 (Pre-COVID) | *(ref)* |  | *-* | *(ref)* |  |  | *(ref)* |  | *-* | *(ref)* |  | *-* |
| Schools Closed | 0.15 | .659 |  | **-0.01** | **.972** |  | -0.03 | .551 |  | 0.40 | .154 |  |
| Schools Reopened | 0.09 | .812 |  | **-0.23** | **.447** |  | -.04 | .281 |  | 0.58 | .055 |  |
|  | **Positive Attitude** | | | | | | **Negative Attitude** | | | | | |
|  | *Male* | | | *Female* | | | *Male* | | | *Female* | | |
|  | Estimate | *p* |  | Estimate | *p* |  | Estimate | *p* |  | Estimate | *p* |  |
| Time - |  |  |  |  |  |  |  |  |  |  |  |  |
| Time 1 (Pre-COVID) | -0.42 | .218 |  | -0.71 | .009 |  | **1.02** | **.004** |  | **0.67** | **.016** |  |
| Time 2 (Pre-COVID) | *(ref)* |  | *-* | *(ref)* |  | *-* | *(ref)* |  | *-* | *(ref)* |  | *-* |
| Schools Closed | 0.93 | .004 |  | 0.26 | .315 |  | 0.15 | .641 |  | 0.23 | .393 |  |
| Schools Reopened | 1.50 | <.001 |  | **1.03** | **<.001** |  | 0.12 | .727 |  | **-0.56** | **.040** |  |

*Note.* Cohen’s d represents change over time relative to Time 2 levels.

**Effect Size of Changes in Symptoms by Initial Severity**

**Supplementary Figure 1.** Effect sizes for changes in symptoms over time compared to Pre-COVID levels.

**Effect Size of Changes in Symptoms by Gender**

**Male**

**Female**

**Supplementary Figure 2.** Effect sizes for changes in symptoms over time compared to Pre-COVID levels.

**Cross-Lagged Panel Models assessing Temporal Relationships between Loneliness and Mental Health**

All standardized model parameters have been presented graphically. Significant effects have been denoted with an asterisk symbol.

**Supplementary Figure 3 – Friendship Quality**


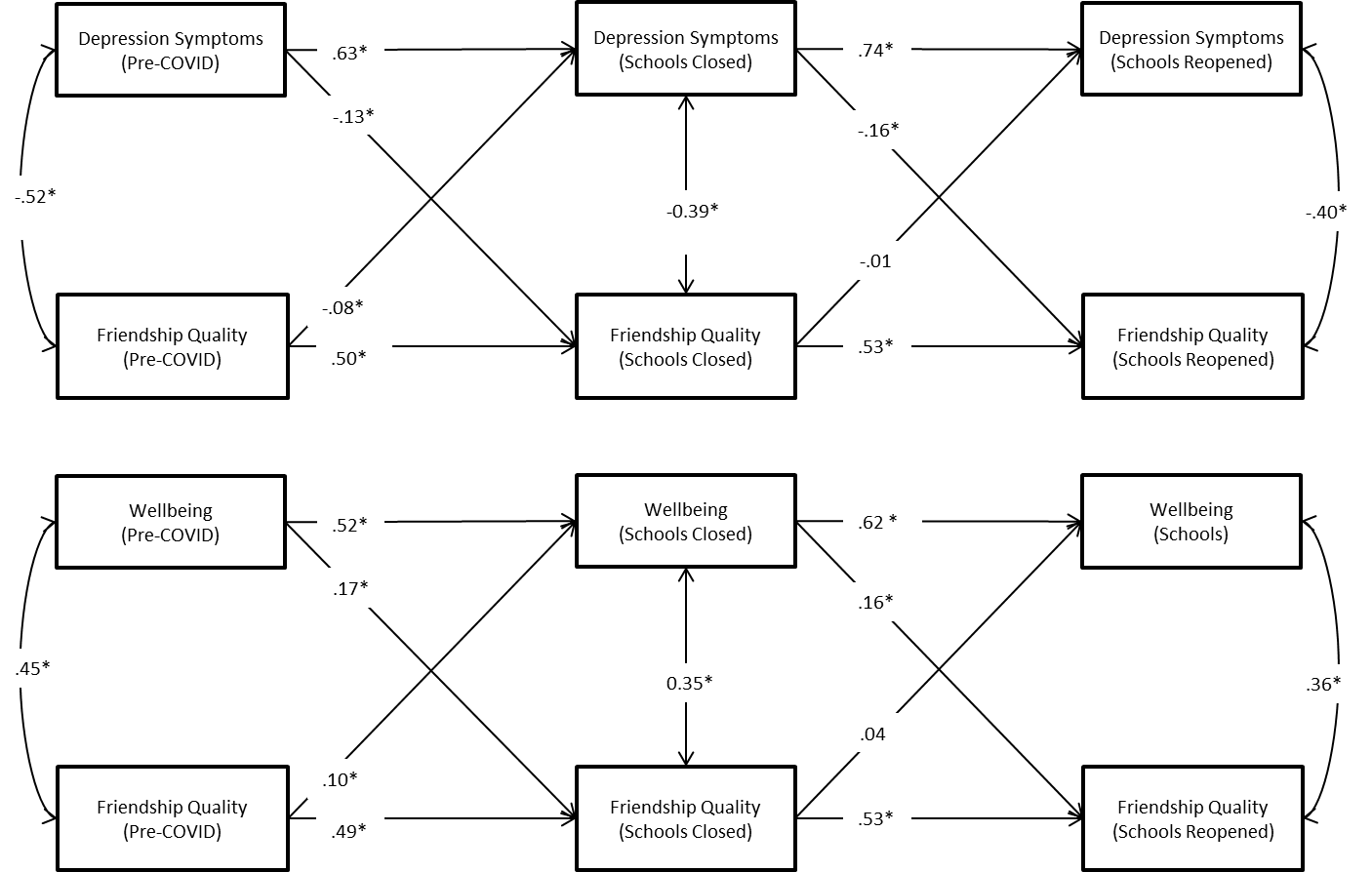


**Supplementary Figure 4 - Isolation**


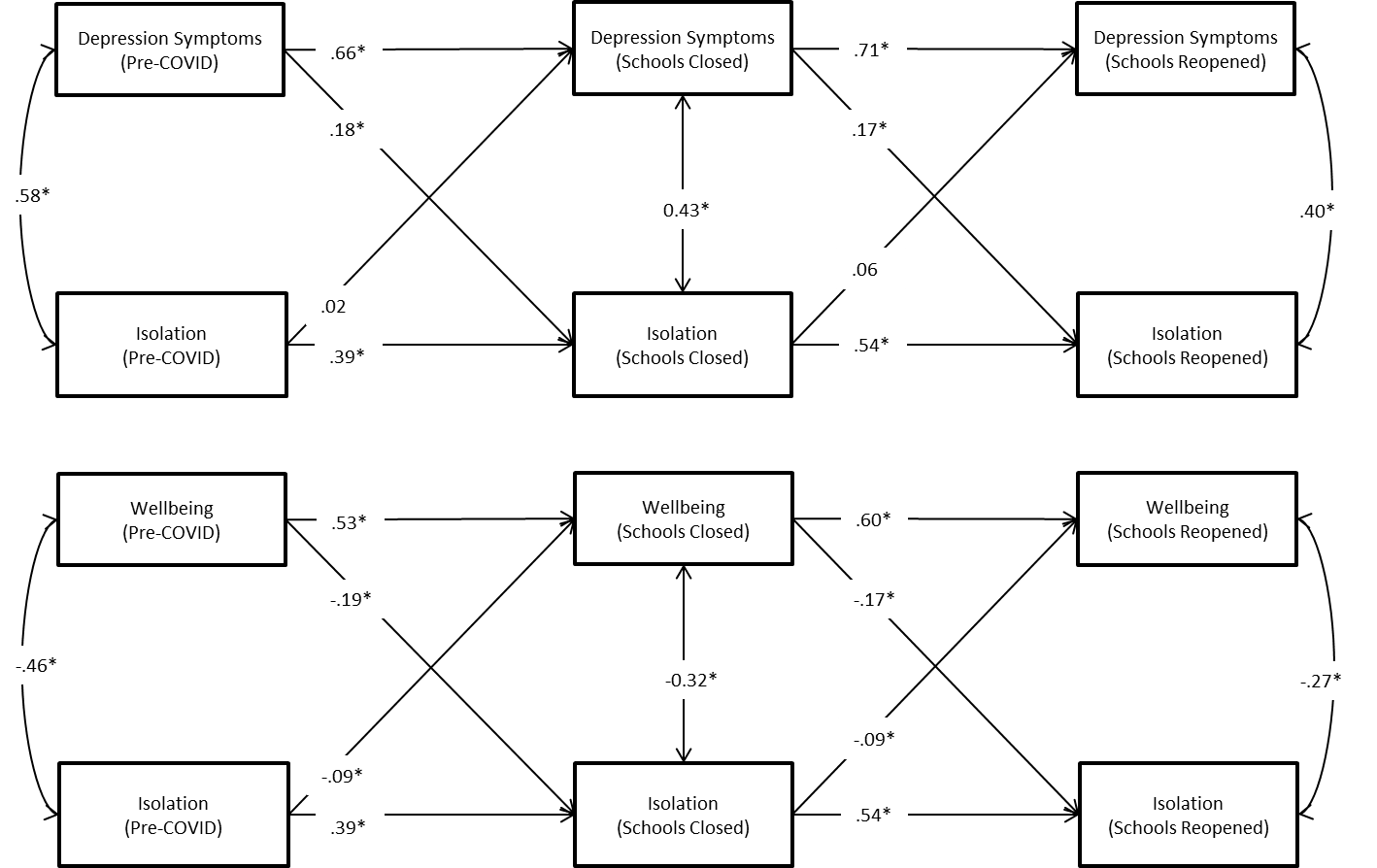


**Supplementary Figure 5 - Positive Attitudes**

**
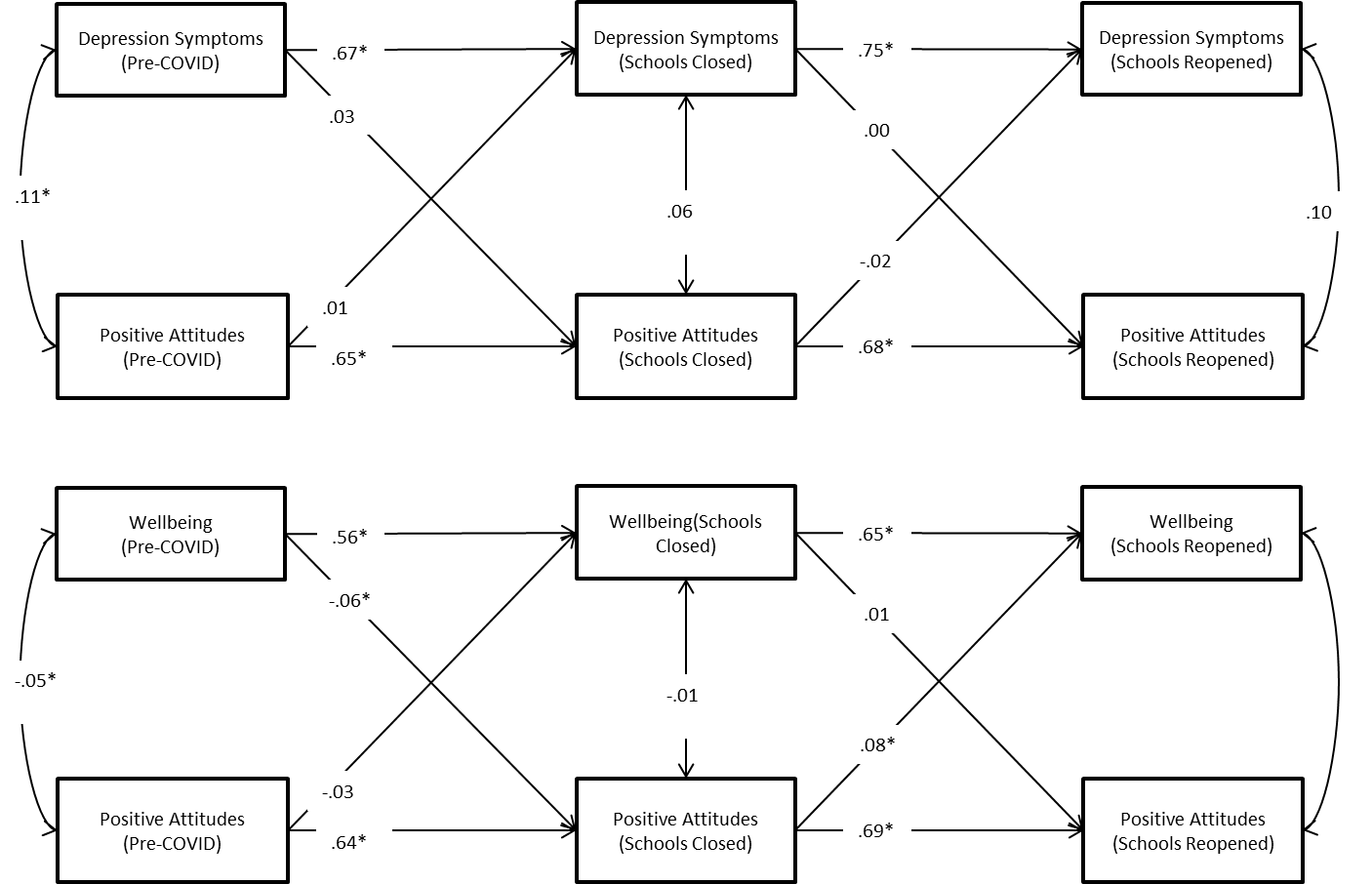
**

**Supplementary Figure 6 - Negative Attitudes**


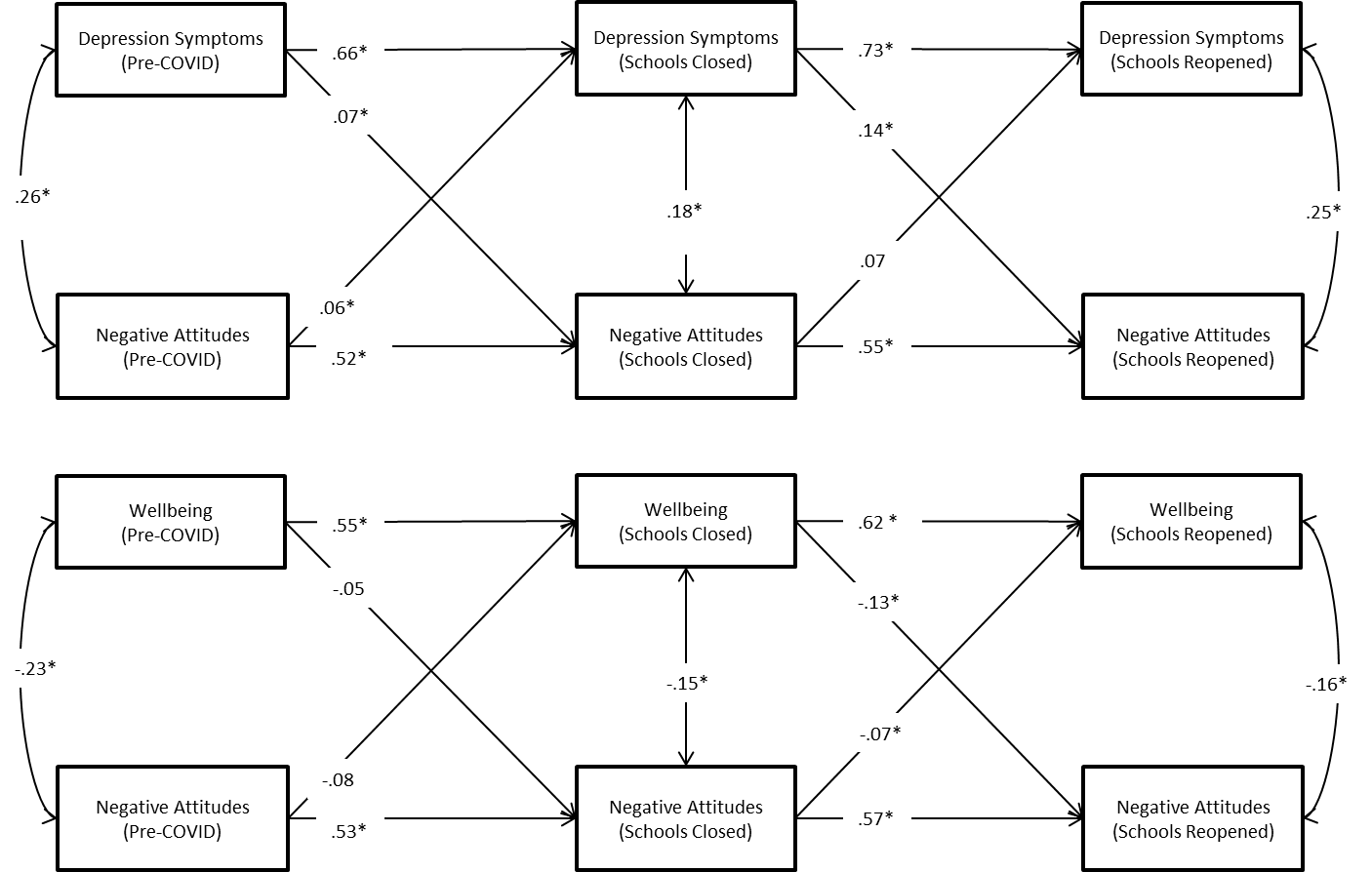


**Final Cross-Lagged Panel Models**

**Supplementary Figure 7 - Depression Symptoms**


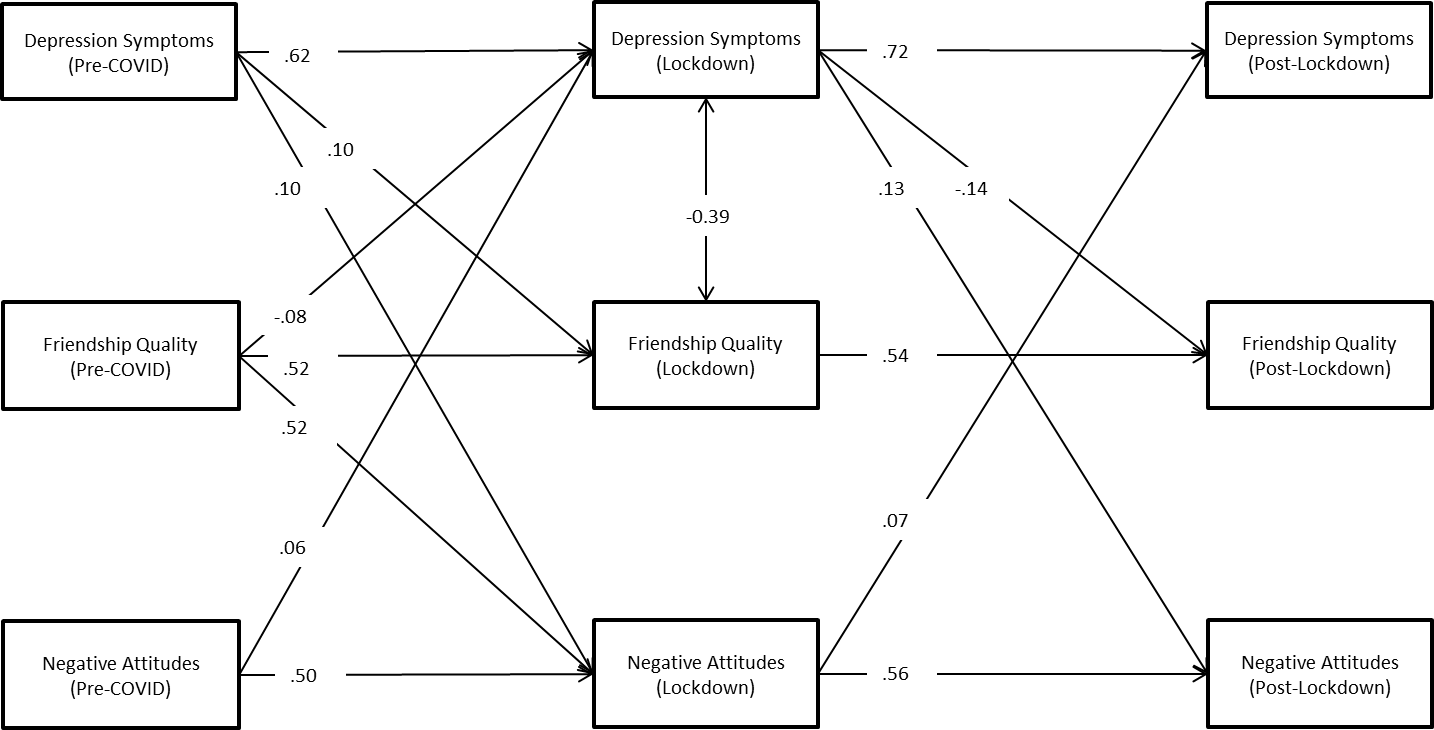


**Supplementary Figure 8 – Positive Mental Wellbeing**

**
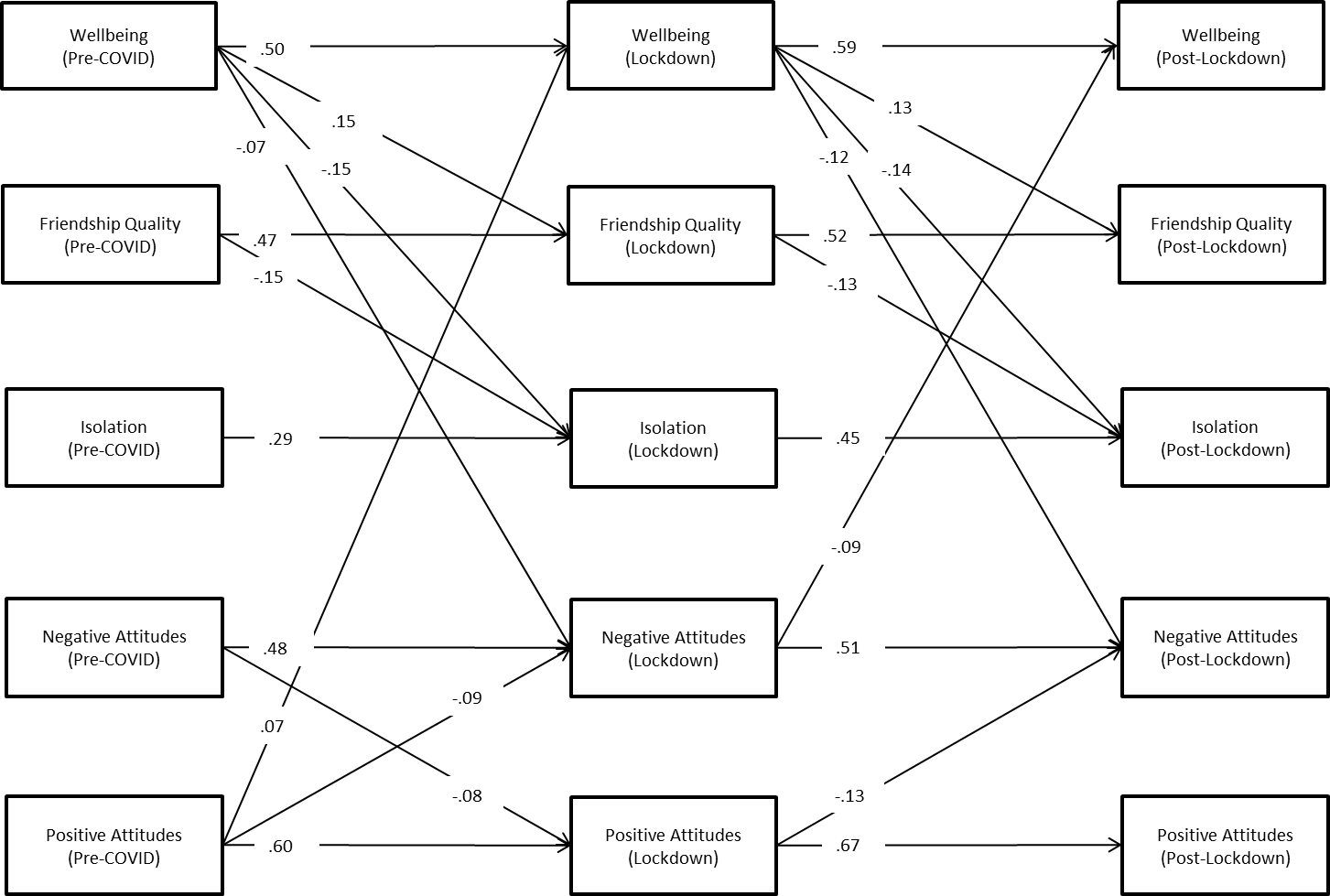
**
